# A *Ralstonia solanacearum* type III effector alters the actin and microtubule cytoskeleton to promote bacterial virulence in plants

**DOI:** 10.1371/journal.ppat.1012814

**Published:** 2024-12-26

**Authors:** Rachel Hiles, Abigail Rogers, Namrata Jaiswal, Weiwei Zhang, Jules Butchacas, Marcus V. Merfa, Taylor Klass, Pragya Barua, Venkatesh P. Thirumalaikumar, Jonathan M. Jacobs, Christopher J. Staiger, Matthew Helm, Anjali S. Iyer-Pascuzzi

**Affiliations:** 1 Department of Botany and Plant Pathology, and Center for Plant Biology, Purdue University, West Lafayette, Indiana, United States of America; 2 EMBRIO Institute, Purdue University, West Lafayette, Indiana, United States of America; 3 Crop Production and Pest Control Research Unit, USDA-ARS: USDA Agricultural Research Service, West Lafayette, Indiana, United States of America; 4 Department of Plant Pathology, The Ohio State University, Columbus, Ohio, United States of America; 5 Bindley Bioscience Center, Purdue University, West Lafayette, Indiana, United States of America; University of Florida Institute of Food and Agricultural Sciences, UNITED STATES OF AMERICA

## Abstract

Cellular responses to biotic stress frequently involve signaling pathways that are conserved across eukaryotes. These pathways include the cytoskeleton, a proteinaceous network that senses external cues at the cell surface and signals to interior cellular components. During biotic stress, dynamic cytoskeletal rearrangements serve as a platform from which early immune-associated processes are organized and activated. Bacterial pathogens of plants and animals use proteins called type III effectors (T3Es) to interfere with host immune signaling, thereby promoting virulence. We previously found that RipU, a T3E from the soilborne phytobacterial pathogen *Ralstonia solanacearum*, co-localizes with the plant cytoskeleton. Here, we show that RipU from *R*. *solanacearum* K60 (RipU^K60^) associated with and altered the organization of both the actin and microtubule cytoskeleton. We found that pharmacological disruption of the tomato (*Solanum lycopersicum*) cytoskeleton promoted *R*. *solanacearum* K60 colonization. Importantly, tomato plants inoculated with *R*. *solanacearum* K60 lacking RipU^K60^ (Δ*ripU*^*K60*^) had reduced wilting symptoms and significantly reduced root colonization when compared to plants inoculated with wild-type *R*. *solanacearum* K60. Collectively, our data suggest that *R*. *solanacearum* K60 uses the type III effector RipU^K60^ to remodel cytoskeletal organization, thereby promoting pathogen virulence.

## Author summary

Pathogenic bacteria secrete dozens of proteins directly into plant and animal cells to interfere with host biology and promote disease. These proteins, termed effectors, target different host proteins to alter cellular processes and enable pathogen virulence. Understanding how distinct effectors manipulate host proteins is critical for developing new disease control strategies. The cytoskeleton is a protein network found across eukaryotic organisms that can sense external signals and transmit responses to the cell interior. Dynamic reorganization of the cytoskeleton is required for immune signaling. Here we showed that the cytoskeleton is critical for resistance to the soilborne phytobacterial pathogen *Ralstonia solanacearum*. We found that RipU, an *R*. *solanacearum* effector protein, physically associates with and alters the organization of the actin and microtubule cytoskeleton in plants. We also found that RipU is required for full pathogen virulence. While an *R*. *solanacearum* mutant that is defective in *ripU* is unable to cause disease at the same level as wild type *R*. *solanacearum*, chemical disruption of the cytoskeleton restored full virulence to these mutants. Our study provides new insights into how pathogenic bacteria manipulate cellular targets to cause disease.

## Introduction

Microbial pathogens use secreted virulence proteins to cause disease in plants and animals. Pathogenic bacteria deliver type III effector proteins (T3Es) into eukaryotic host cells where they subsequently interact with and manipulate host proteins to suppress a diverse range of processes, including immune signaling [1–3]. The cytoskeleton is an intracellular filamentous network that is essential for cellular homeostasis and is a critical part of immune signaling in eukaryotes [4–7]. Several T3Es from plant and animal pathogenic bacteria interact directly or indirectly with components of the cytoskeleton and suppress immune responses [1,7–10]. The underlying cellular mechanisms for how pathogen-derived effectors manipulate the cytoskeleton, how such manipulation interferes with immune signaling, and how this promotes pathogen virulence remain largely unknown. Knowledge as to how pathogenic bacteria manipulate cellular targets will provide insight into host-microbe cellular biology as well as contribute to our understanding of putative disease control strategies [11,12].

*Ralstonia solanacearum* (*R*. *solanacearum*) is a soil-borne phytopathogen that causes bacterial wilt disease in over 250 plant species, including economically and agriculturally important cash crops such as potato (*Solanum tuberosum*), pepper (*Capsicum annum*), and tomato (*Solanum lycopersicum*) [13–17]. Similar to other pathogenic bacteria, *R*. *solanacearum* utilizes T3E proteins to promote its virulence, but host targets of this pathogen are not well defined [18,19]. In most crop species, host genetic resistance to *R*. *solanacearum* is a quantitative trait and relies upon the action of several genomic regions known as quantitative trait loci (QTL) [17,20–22]. Despite the importance of *R*. *solanacearum* worldwide, mechanisms underlying this quantitative resistance remain largely unknown, but likely involve developmental and basal immune processes [13,16,23].

Immune signaling pathways and their associated proteins are frequent targets of effectors in plants and animals. During early invasion by microbes, eukaryotic host cells recognize Microbe-Associated Molecular Patterns (MAMPs) through cognate cell surface receptors. MAMP perception elicits a set of rapid signaling events, including production of reactive oxygen species (ROS), calcium (Ca^2+^) influx, phospholipid fluxes, and Mitogen Activated Protein Kinase (MAPK) phosphorylation, which ultimately lead to changes in host defense gene transcription and collectively are known as pattern-triggered immunity (PTI) [24]. The cytoskeleton is an essential signaling intermediate during PTI [4,5,7]. This intracellular filamentous network controls cell shape and division, organellar movement, endocytosis and secretion, and provides the tracks for intracellular and intercellular trafficking. In plants, the cytoskeleton is composed of two major filament systems, actin filaments and microtubules, along with many additional accessory proteins that are required for cytoskeletal function [25]. During immune signaling in both plants and animals, the actin cytoskeleton promotes antimicrobial protein transport, facilitates immune receptor dynamics at the plasma membrane, and is dynamically linked to ROS burst, phospholipid signaling and Ca^2+^ fluxes [4,5,7,26–28]. In both plants and animals, inhibiting cytoskeleton dynamics with either genetic mutants or pharmacological inhibitors can promote pathogen virulence [27,29–31].

Both actin and microtubules transiently polymerize and depolymerize in response to internal and external stimuli, including beneficial and pathogenic microbes [4,5,7]. For example, fungal and oomycete invasion promotes actin filament accumulation at the attempted point of penetration [32–35]. Additionally, a transient increase in the density of cortical actin filaments occurs within minutes after bacterial MAMP perception in Arabidopsis (*Arabidopsis thaliana*) and is an early hallmark of PTI [29,36,37]. Arabidopsis mutants defective in early actin remodeling or dynamics are more susceptible to the bacterial pathogen *Pseudomonas syringae* pv. *tomato* DC3000 (*Pst* DC3000) [29] and have altered immune outputs [36–40]. Unlike changes to actin organization, significant changes in microtubule organization in response to MAMPs or pathogen perception are less well characterized. Microtubule reorganization occurs in response to both beneficial fungi and during pathogen invasion [41,42]; however, the detailed role of microtubules in plant immunity has not been elucidated.

Although the underlying cellular mechanisms for how pathogen-derived effectors manipulate the cytoskeleton, how this promotes pathogen virulence remain largely unknown. Nevertheless, several T3Es from phytopathogenic bacteria have been identified that target either the actin or microtubule cytoskeleton. For example, the *Pseudomonas syringae* effectors HopW1 and HopG1 [30,43,44] as well as XopR from *Xanthomonas campestris* [39] alter actin structure and organization either by directly interacting with actin filaments (HopW1) or by interfering with actin-associated proteins (XopR and HopG1). Additional T3Es impact microtubules either directly or indirectly. XopL from *X*. *euvesicatoria* directly interacts with microtubules and causes cell death when transiently expressed in *N*. *benthamiana* [45]. Transient expression of HopZa1 from *Pseudomonas syringae* causes destruction of microtubule networks, inhibits protein secretion, and suppresses cell wall-mediated defenses [31]. The T3Es HopE1 and AvrBsT indirectly impact microtubule organization by interfering with microtubule associated proteins MAP65 [46] and ACIP1 [47], respectively.

We previously showed that RipU^K60^, a T3E from *R*. *solanacearum* strain K60, qualitatively co-localizes with the actin cytoskeleton in tomato roots and leaves of *N*. *benthamiana*, suggesting it may have a functional role in cytoskeleton-mediated immune signaling [19]. Here, using high-resolution spinning disk confocal microscopy (SDCM) and quantitative image analysis, we demonstrate that RipU^K60^ alters the organization of both the actin and microtubule cytoskeleton. We found that RipU^K60^ physically associated with both tubulin and actin. Cytoskeleton disruption using the pharmacological inhibitors LatrunculinB (LatB; actin) or oryzalin (microtubules) promoted *R*. *solanacearum* colonization in tomato roots, demonstrating that the cytoskeleton has a functional role in *R*. *solanacearum* recognition. A *R*. *solanacearum* mutant lacking RipU^K60^ had decreased virulence and colonization in naturalistic soil drench assays. Collectively, our data suggest that RipU^K60^ promotes *R*. *solanacearum* virulence likely by associating and interfering with the dynamics and organization of both the actin and microtubule cytoskeleton.

## Results

### An *R. solanacearum* Type III effector co-localizes with the cytoskeleton

**Fig 1:**
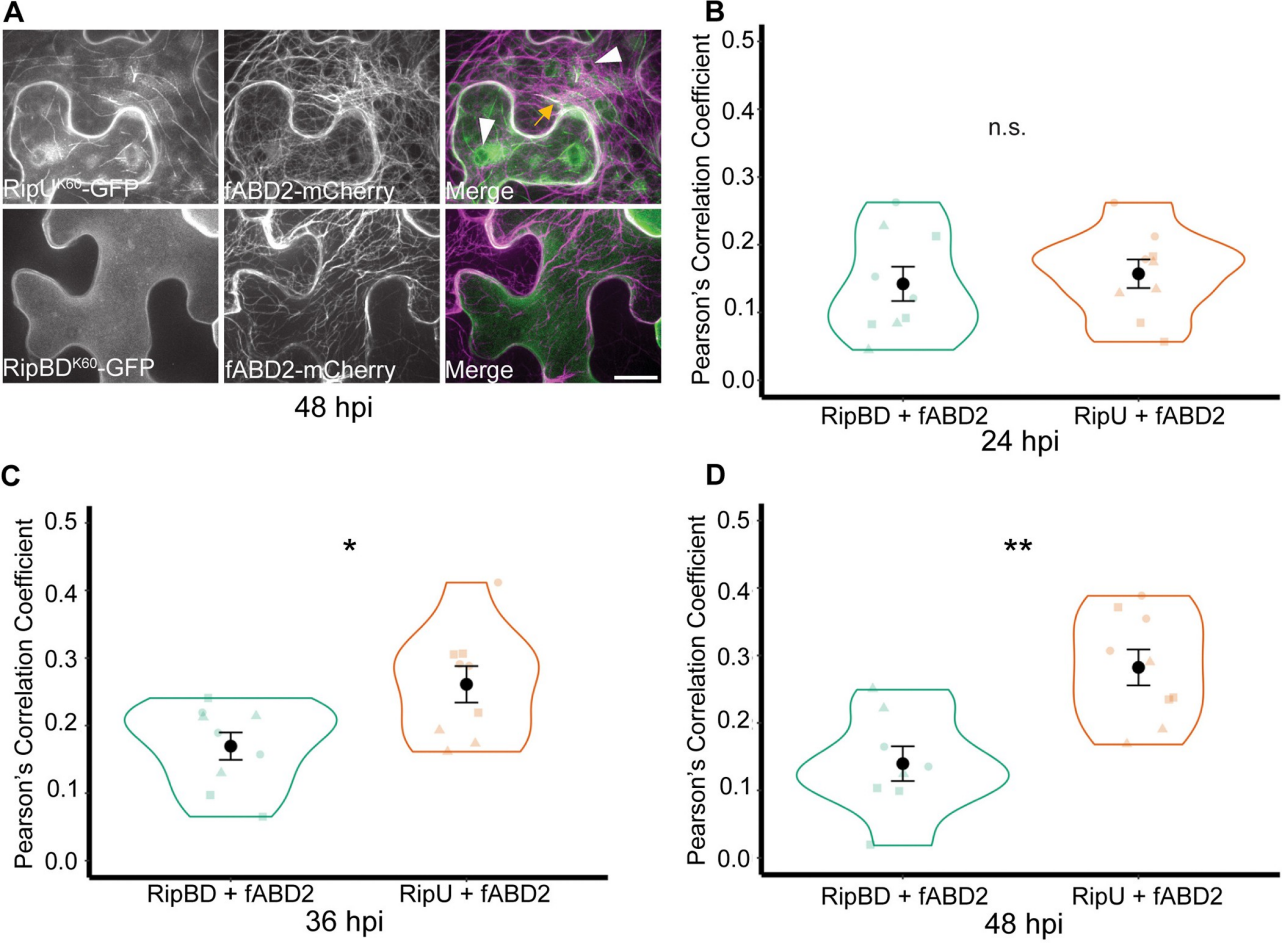
Transient expression of RipU^K60^ with the actin reporter fABD2-mCherry at 24, 36 and 48 hours post infiltration. (A) RipU^K60^-GFP (top panel) or RipBD^K60^-GFP (bottom panel) were co-infiltrated with the actin marker fABD2-mCherry in *N*. *benthamiana* leaves. Representative images taken at 48 hours post infiltration (hpi). Orange arrow points to overlap of filamentous structures of RipU^K60^-GFP with fABD2-mCherry. RipU and fABD2 appear to surround an intracellular compartment (white arrows). (B-D) Co-localization was quantified at 24 (B), 36 (C) and 48 (D) hours post infiltration (hpi) using Pearson’s correlation coefficient analysis (Pcc). RipU^K60^ significantly co-localizes with the actin cytoskeleton compared to RipBD^K60^ at 36 and 48 hpi. Five to fifteen cells were measured at each infiltration site and the values were averaged as one biological sample (n). Three biological samples were quantified in each of three independent experiments. Each independent experiment is depicted as a different shape within each treatment. T test; *P < 0.05, ** P < 0.01. Scale bar = 20 μm.

Using laser scanning confocal microscopy we previously showed that the *R*. *solanacearum* K60 T3E RipU^K60^ qualitatively co-localized with the actin marker fABD2-mCherry in tomato hairy roots and *N*. *benthamiana* leaves [19]. To quantify this co-localization and extend this analysis to the microtubule cytoskeleton, we investigated the subcellular localization of RipU^K60^-GFP using spinning disk confocal microscopy (SDCM) following transient co-expression in *N*. *benthamiana* leaves with cytoskeletal reporters. We first confirmed that RipU^K60^ is secreted through the type III secretion system (S1 Fig), similar to RipU from strains *R*. *pseudosolanacearum* GMI1000 [48] and *R*. *solanacearum* strain P380 [49]. We next imaged and quantified the subcellular co-localization of RipU^K60^-GFP with either the actin reporter fABD2-mCherry or the microtubule reporter TUB5-mCherry. Transient expression of RipU^K60^-GFP did not cause any visual changes to the *N*. *benthamiana* leaf by 72 hpi (S2 Fig). GFP-tagged RipU^K60^ and fABD2-mCherry were expressed in *N*. *benthamiana* leaves using *Agrobacterium tumefaciens*-mediated transient transformation (agroinfiltration) and imaged using SDCM at 24-, 36- and 48-hours post-agroinfiltration (hpi) (Figs 1 and S3). As a negative control we included another *R*. *solanacearum* K60 T3E, RipBD^K60^-GFP, that localizes to the plasma membrane and did not demonstrate significant co-localization with fABD2 (Fig 1; Pearson’s Correlation Coefficient (Pcc) values < 0.15). At 24 hpi, the colocalization of RipU^K60^ with fABD2-mCherry did not significantly differ from the colocalization of the negative control RipBD^K60^ (Fig 1B; t-test, p-value = 0.66). However, at 36 hpi RipU^K60^ colocalization with fABD2-mCherry was small (Pcc = 0.26) but significantly greater than the negative control RipBD^K60^ (Fig 1B; t-test p-value = 0.015), and this was maintained at 48 hpi (Fig 1C; Pcc = 0.28, p-value = 0.0016 in t-test vs RipBD^K60^). Curiously, in *N*. *benthamiana* cells expressing both RipU^K60^-GFP and fABD2-mCherry at 24-, 36- and 48-hours, we observed that both RipU^K60^ and fABD2 appeared to surround an intracellular component (Figs 1A and S3).

**Fig 2:**
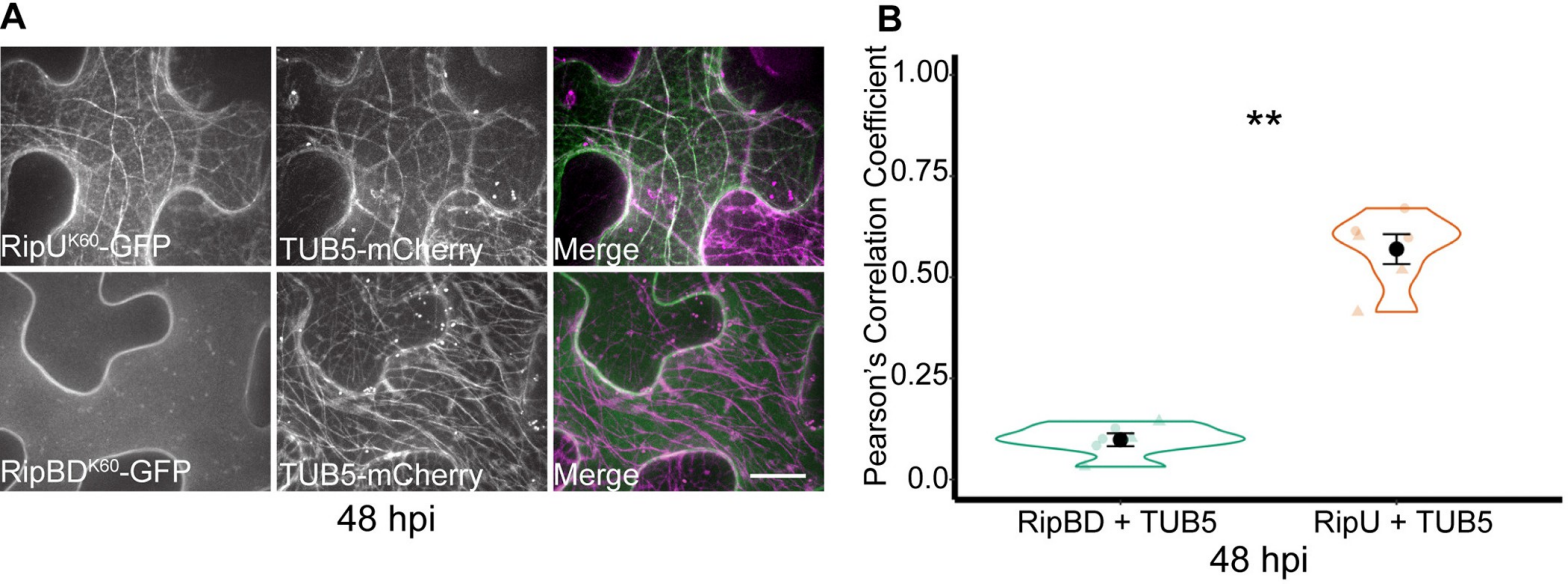
RipU^K60^ colocalizes with the plant microtubule cytoskeleton at 48 hours post infiltration. RipU^K60^-GFP or RipBD^K60^-GFP were co-infiltrated with the microtubule marker TUB5-mCherry in *N*. *benthamiana* leaves. (A) Representative images demonstrate the co-localization of RipU^K60^-GFP with TUB5-mCherry. (B) Co-localization was quantified at 48 hpi using Pcc analysis. At 48 hpi, RipU^K60^ significantly co-localized with microtubules compared to RipBD^K60^. Five to fifteen cells were measured at each infiltration site and the values were averaged as one biological sample (n). Three biological samples were quantified in each of two independent experiments. Each independent experiment is depicted as a different shape within each treatment. Wilcoxon rank sum test; *P<0.05, ** P < 0.01. Scale bar = 20 μm.

Transient expression of RipU^K60^ with the microtubule reporter TUB5-mCherry revealed co-localization at 48 hpi (Fig 2; Pcc = 0.57,Wilcoxon rank sum test, p-value = 0.0051). As with fABD2, GFP-tagged RipBD^K60^ did not significantly co-localize with TUB5-mCherry (Fig 2B). Thus, RipU^K60^ appears to moderately co-localize with TUB5-mCherry at 48 hpi.

It is possible that our observed co-localization with fABD2 and TUB5 was indirect, or was due to high levels of RipU^K60^-GFP fluorescence in the cortical cytoplasm. To examine this, we repeated our co-localization experiments with RipAY^GMI^-GFP, an effector protein from *R*. *pseudosolanacearum* strain GMI1000 that localizes to the cytoplasm and nucleus [50]. Because of the density of RipAY^GMI^-GFP in the cortical cytoplasm that was observed with maximum projections of SDCM images (S4 Fig), we used only the uppermost two slices of z-stacks for the analysis. Although co-localization of RipU^K60^-GFP with the actin reporter fABD2-GFP was significantly greater than co-localization of RipBD-GFP, it was not significantly greater than the cytoplasmic effector RipAY^GMI^-GFP (p < 0.2; S5A and S5C Fig). Quantification of co-localization of RipU and fABD2 in the uppermost two slices of the z-stack revealed a low Pcc value (Pcc < 0.2). However, overlap of filamentous structures of RipU^K60^-GFP and fABD2-GFP was occasionally observed (arrows, S5A Fig). In contrast, such overlap was not observed when RipAY^GMI^-GFP was co-expressed with fABD2-mCherry (S5A Fig). Transient expression of RipU^K60^-GFP with the microtubule reporter TUB5-mCherry revealed a moderate co-localization value of Pcc = 0.55 at 48 hpi (S5B and S5D Fig), significantly greater than the Pcc value for RipAY^GMI^-GFP (Pcc = 0.36) and RipBD^K60^-GFP (Pcc = 0.13). Together, these data show that RipU^K60^-GFP co-localizes with microtubules in *N*. *benthamiana* leaves but may not co-localize with actin filaments in *N*. *benthamiana* cells. However, the close proximity of some filamentous structures of RipU^K60^-GFP (S5A Fig, arrows) to fABD2-mCherry suggests that there may be an association between RipU^K60^-GFP and both components of the cytoskeleton.

### RipU^K60^ associates with actin and tubulin

**Fig 3:**
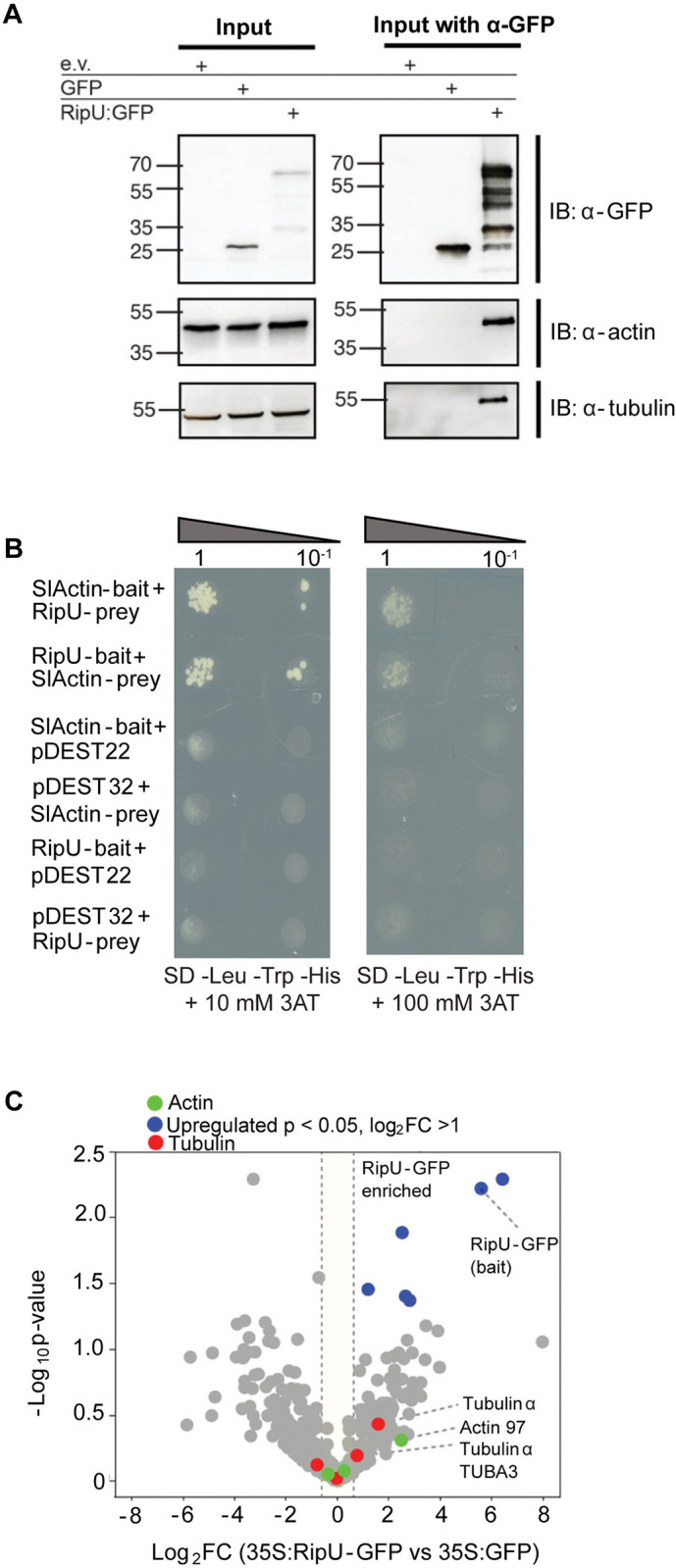
RipU^K60^ physically associates with the cytoskeleton. (A) RipU^K60^-GFP co-immunoprecipitates with actin and tubulin. The indicated constructs were transiently expressed in *N*. *benthamiana* leaves. All transgenes were under the control of a 35S promoter. Total protein was isolated 48 hpi, immunoprecipitated by GFP-Trap agarose bead slurry, and immunoblotted with the indicated antibodies. (B) RipU^K60^ associates with tomato actin in a yeast two hybrid assay. Left, SC-Leu-Trp-His + 10 mM 3AT selection plates, right SC-Leu-Trp-His + 100 mM 3AT. Top two rows of each set of panels show that *Sl*Actin and RipU interaction promotes yeast growth when cloned as either bait (pDEST32) or prey (pDEST22). Four lower rows are negative controls with either *Sl*Actin or RipU and an empty bait or prey vector. Dilutions of indicated constructs were plated on yeast selective media (SC-Leu-Trp-His +3AT). All experiments were repeated at least three independent times. (C) Co-IP/MS analysis. Immunoprecipitation was done by GFP-Trap agarose bead slurry, and eluants were subjected to mass spectrometry. The volcano plots depict the differential enrichment of proteins between *35S*:*RipU*^*K60*^ *-GFP* and *35S*:*GFP*. The blue dots are proteins with p-value ≤ 0.05 and log_2_FC > 1. Actin and tubulin proteins are indicated in green and red respectively. Two tubulin alpha proteins and an actin protein with greater than 1.5-fold change (log_2_FC > 0.585) are indicated. The result represents three independent experiments.

The spinning disk confocal microscopy results suggested that RipU^K60^ may physically interact with the plant cytoskeleton. To investigate whether RipU^K60^ associates with actin and tubulin, we transiently expressed RipU^K60^-GFP and performed coimmunoprecipitation (co-IP) assays. Consistent with our hypothesis, both actin and tubulin were detected in the RipU^K60^-GFP immunoprecipitates (Fig 3A). As a control, we transiently expressed free GFP, which did not immunoprecipitate actin or tubulin (Fig 3A).

For yeast two-hybrid assays, we cloned a gene encoding another tomato actin isoform (*SlActin*: *Solyc10g086460*; over 95% similar to *At5g59370*, Arabidopsis *Actin4*) and RipU into bait (pDEST32) and prey (pDEST22) plasmids. We spotted dilutions onto auxotrophic selective plates (Fig 3B). Yeast growth was observed for both reciprocal combinations of actin and RipU (*Sl*ACT bait + RipU prey and RipU bait + SlACT prey)(Fig 3B). Growth was higher on selection plates containing lower levels of 3AT (10 mM), a competitive inhibitor of HIS3, but was still present when the gene was inhibited with 100 mM 3AT. Yeast growth was not observed when either SlActin or RipU was co-expressed with an empty bait or prey vector (Fig 3B).

For co-immunoprecipitation (Co-IP), three independent experiments were conducted, followed by liquid chromatography-tandem mass spectrometry (LC-MS/MS) analysis. *35S*:*RipU*^*K60*^ *–GFP* and *35S*:*GFP* (negative control) were transiently expressed in *N*. *benthamiana* leaves using agroinfiltration. We also included *35S*:*RipAM*^*K60*^, an effector that localizes to the nucleus [19]. Tissue was harvested 48 hours after infiltration. The eluates from samples (RipU^K60^, RipAM^K60^ or GFP) in each experiment were subjected to untargeted proteomics via LC-MS/MS. Principal component analysis (PCA) demonstrated a distinct separation between the *35S*:*RipU*^*K60*^*—GFP* and *35S*:*GFP* samples, with experimental variation likely contributing to a reduction in *p*-values for weaker interactors (S6 Fig). As anticipated, the bait protein RipU^K60^ was highly abundant (Fig 3C, top right quadrant). Normalized abundances within each sample revealed the presence of two actin proteins, an actin binding protein known as actin-depolymerizing factor 2, two alpha tubulin and two beta tubulin proteins in the RipU and control (GFP, RipAM) samples. However, an alpha tubulin, beta tubulin and the actin depolymerizing protein were identified in all three experiments only for RipU (S1 Table). Further, a 1.5-fold (log_2_FC > 0.585) or more enrichment of two tubulin alpha subunits and one actin subunit was observed in the RipU samples compared to GFP only samples (Fig 3C, labeled dots and S1 Table). These data suggest an association of RipU^K60^ with cytoskeletal subunits using untargeted approaches *in-planta*.

Collectively, all of these results demonstrate that RipU^K60^ physically associates with components of the plant cytoskeleton.

### RipU^K60^ contributes to pathogenesis and virulence

**Fig 4:**
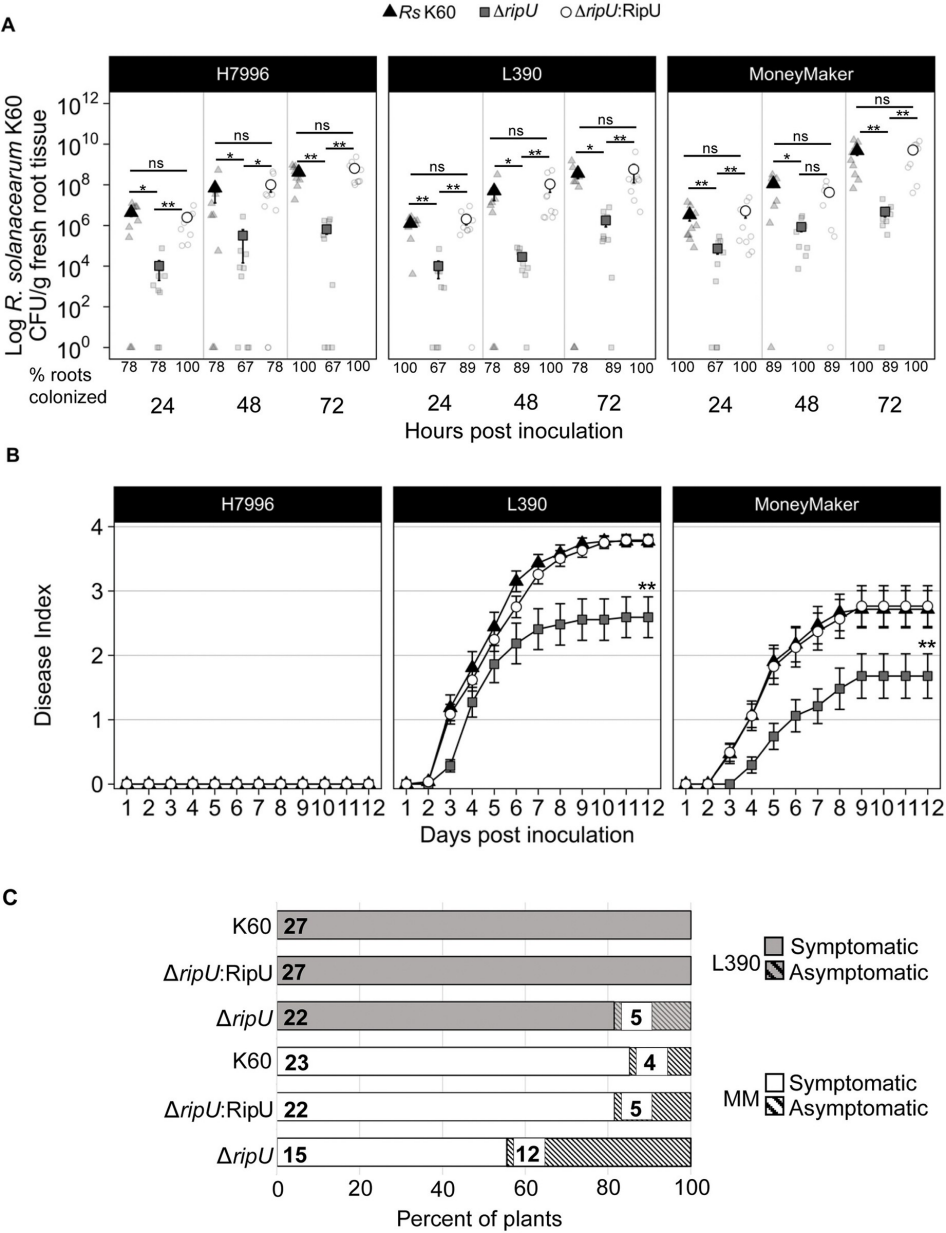
RipU^K60^ is required for full *R*. *solanacearum* K60 virulence in tomato. (A) Root colonization of *R*. *solanacearum* K60, Δ*ripU*^*K60*^ and Δ*ripU*^*K60*^::*RipU*^*K60*^ in whole roots of H7996 (resistant), L390 (susceptible) and MM (susceptible). Three independent experimental replicates were performed. Each replicate consisted of three individual roots per timepoint and genotype for a total of nine plants for each genotype, timepoint and treatment across all replicates. Each dot represents one root (n = 9 per timepoint and genotype). Stars indicate significance with a Wilcoxon test (* = P < 0.05, ** = P < 0.01, *** = P < 0.001). Error bars indicate standard deviation. (B and C) Infection with Δ*ripU*^*K60*^ results in less wilting symptoms in susceptible varieties. (B) Average wilting scores of inoculated plants. The Δ*ripU*^*K6*0^ mutant has delayed symptom development and less wilting at 12 dpi compared to either wild-type *R*. *solanacearum* K60 or Δ*ripU*^*K60*^::*RipU*^*K60*^. Wilting was scored daily based on the number of leaves wilted per plant. Each point represents the average of 3 independent experiments, each with 12 plants per genotype per treatment. Stars indicate that the AUDPC is significantly different (P < 0.01, t-test) between wild-type *R*. *solanacearum* K60 and Δ*ripU*^*K60*^ and between the complemented strain Δ*ripU*^*K60*^::*RipU*^*K60*^ and Δ*ripU*^*K60*^ for both MM and L390. (C) Percent of plants showing wilting symptoms. Fewer wilt-susceptible L390 and MM tomato plants showed wilting symptoms when soil-drench inoculated with Δ*ripU*^*K60*^ mutant strain compared to either wild-type *R*. *solanacearum* K60 or the complemented strain Δ*ripU*^*K60*^::*RipU*^*K60*^. MM inoculated with any *R*. *solanacearum* strain reached the highest disease incidence (percent of plants with symptoms) at 7 dpi. L390 inoculated with wild type *R*. *solanacearum* K60 and the complemented strain reached highest disease incidence at 6 dpi.

Given our findings that RipU^K60^ associates with the cytoskeleton and previous reports demonstrating the role of the cytoskeleton in immune signaling, we hypothesized that RipU^K60^ is required for full *R*. *solanacearum* K60 virulence. To test this, we generated a *ΔripU*^*K60*^ single deletion mutant as well as a complemented *R*. *solanacearum* K60 strain Δ*ripU* miniTn*7*::*ripU*K60 (hereafter *ΔripU*^*K60*^::*RipU*^*K60*^). We first compared colonization rates of wild-type *R*. *solanacearum* K60, *ΔripU*^*K60*^, and *ΔripU*^*K60*^::*RipU*^*K60*^ in resistant and susceptible tomatoes. We inoculated three *Solanum lycopersicum* tomato varieties: wilt-resistant Hawaii7996 (H7996), wilt-susceptible L390, and moderately wilt-susceptible Moneymaker (MM) via soil drench inoculation [20,51]. Although H7996 is wilt-resistant, *Ralstonia* is still able to colonize this variety, albeit at lower levels than wilt-susceptible plants [51,52]. *R*. *solanacearum* K60 colonization in roots was quantified at 24, 48 and 72 hpi. In all tomato varieties, *ΔripU*^*K60*^ had significantly lower rates of colonization when compared to both wild-type *R*. *solanacearum* K60 and *ΔripU*::*RipU*^*K60*^ at all time points (~10^4^ CFU/g root tissue compared to ~10^7^ CFU/g root tissue for wild type or complemented strain, Fig 4A). Colonization rates of *ΔripU* ^*K60*^::*RipU*^*K60*^ mimicked those of WT *R*. *solanacearum* K60 and were not significantly different at any time point in any tomato variety (Fig 4A). These data indicate that a functional RipU^K60^ is required for full *R*. *solanacearum* K60 virulence in tomato roots.

Since the absence of RipU^K60^ influenced the ability of *R*. *solanacearum* K60 to colonize tomato roots, we next asked whether RipU^K60^ was required for *R*. *solanacearum* K60 pathogenicity. Wilt-resistant H7996 and wilt-susceptible MM and L390 plants were inoculated with either *R*. *solanacearum* K60, *ΔripU*^*K60*^, or *ΔripU*^*K60*^::*RipU*^*K60*^. Wilting was assayed on a scale of 0 to 4 (no discernable wilting = 0 and 100% of leaves wilted = 4). As expected, wilt-resistant H7996 plants inoculated with wild-type *R*. *solanacearum* K60, *ΔripU*^*K60*^, or *ΔripU* ^*K60*^::*RipU*^*K60*^ did not display any observable wilting symptoms (score of 0 at 12 days post inoculation (dpi); Fig 4B). Wilt-susceptible L390 plants inoculated with the *ΔripU* ^*K60*^ mutant displayed reduced wilting compared to plants inoculated with either the wild type or complemented strain. L390 inoculated with the *ΔripU* ^*K60*^ mutant an average wilting score of 2.6 at 12 dpi and Area Under the Disease Progress Curve (AUDPC) score of 19.5. In contrast, L390 tomatoes inoculated with wild-type *R*. *solanacearum* K60 had average wilting scores of 3.9 and AUDPC 28.8 while those inoculated with *ΔripU* ^*K60*^::*RipU*^*K60*^ showed average wilting score of 3.8 and AUDPC of 27.6 at 12 dpi (Fig 4B). The AUDPC for wilt-susceptible L390 plants inoculated with *R*. *solanacearum ΔripU*^*K60*^ was significantly lower than the AUDPC for L390 inoculated with either wild-type *R*. *solanacearum* K60 (P = 0.001, one-tailed t-test) or the complemented strain *ΔripU* ^*K60*^::*RipU*^*K60*^ (P = 0.003, one-tailed t-test). Similarly, wilt-susceptible MM plants inoculated with the *ΔripU*^*K60*^ mutant displayed reduced wilting severity (average wilting score of 1.67 at 12 dpi and AUDPC = 10.7; Fig 4C) compared to MM plants inoculated with wild-type *R*. *solanacearum* K60 (average wilting score = 2.7 and AUDPC = 20.2 or *ΔripU* ^*K60*^::*RipU*^*K60*^ (average wilting score = 2.7, AUDPC = 20.1; Fig 4C). The AUDPC for wilt-susceptible MM inoculated with *R*. *solanacearum ΔripU*^*K60*^ was significantly lower than the AUDPC for MM inoculated with either wild-type *R*. *solanacearum* K60 (P = 0.002, one-tailed t-test) or the complemented strain *ΔripU* ^*K60*^::*RipU*^*K60*^ (P = 0.003, one-tailed t-test).

Analysis of disease incidence (number of plants with any symptoms/number of total plants) showed that the disease incidence of both wilt-susceptible genotypes L390 and MM was lower when inoculated with the *ΔripU*^*K60*^ mutant compared to either wild type *R*. *solanacearum* K60 or the complemented *R*. *solanacearum* strain (Fig 4C). L390 plants treated with *R*. *solanacearum* K60 or the complemented *ΔripU* ^*K60*^::*RipU*^*K60*^ reached 100% disease incidence (27/27 plants) at 6 days after inoculation. In contrast, the highest disease incidence for L390 plants treated with the *ΔripU*^*K60*^ mutant was 81.4% (22/27 plants) at 8 dpi (Fig 4C). MM plants inoculated with any strain reached their highest disease incidence at 7 dpi. However, 85% (23/27) of MM treated with *R*. *solanacearum* K60 and 81.4% (22/27) of MM plants inoculated with the complemented strain showed wilting symptoms. In contrast, only 55.5% (15/27) of MM plants inoculated with the *ΔripU*^*K60*^ mutant had wilting symptoms (Fig 4C). Taken together, our findings demonstrate that RipU^K60^ is essential for full pathogenicity and virulence of *R*. *solanacearum* K60.

### Chemical disruption of the actin or microtubule cytoskeleton improves *R. solanacearum* K60 colonization

**Fig 5:**
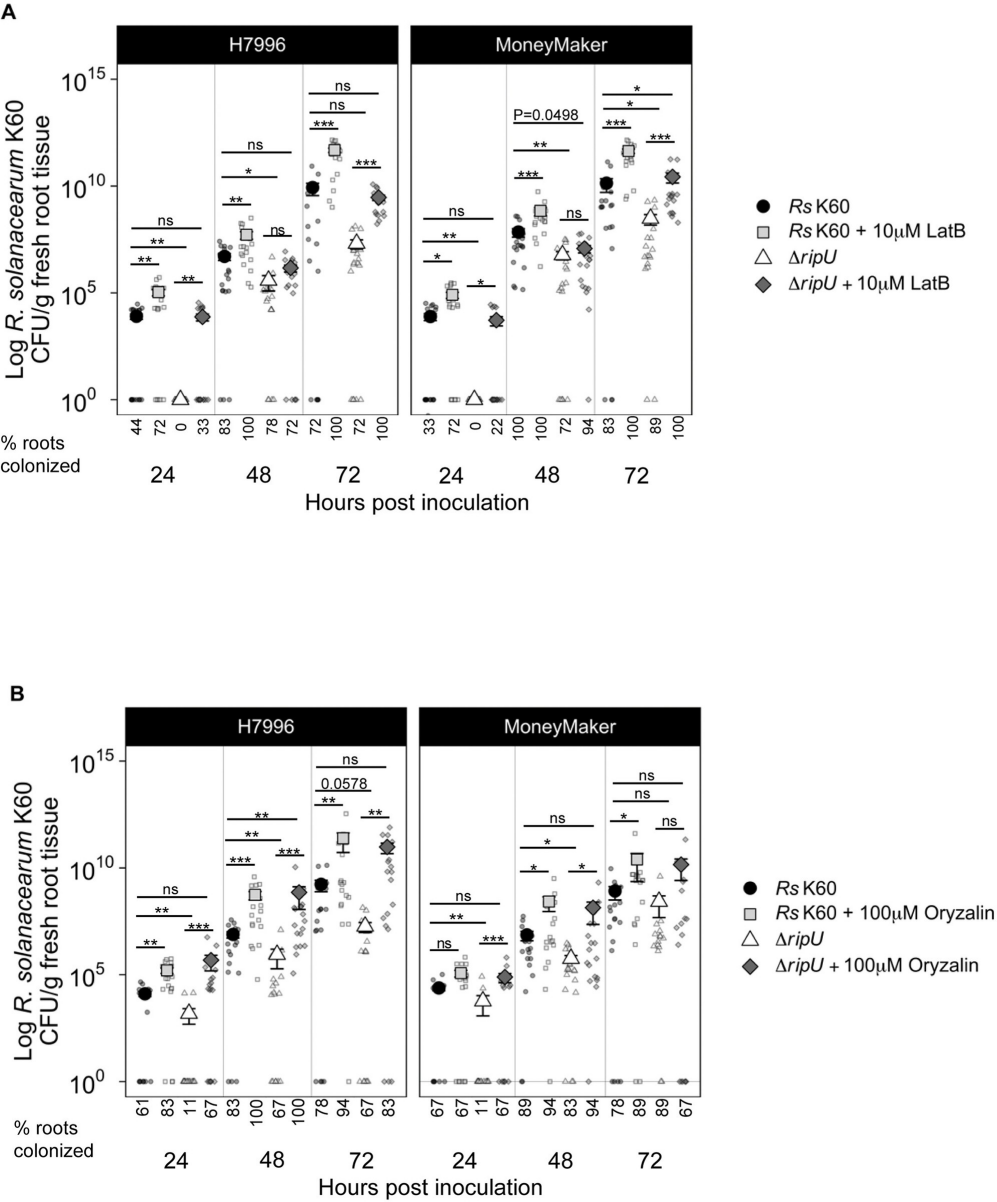
Cytoskeleton disruption improves *R*. *solanacearum* root colonization in both resistant (H7996) and susceptible (Moneymaker) tomato plants. Tomato seedlings were grown on water agar and treated with 10 μM latrunculin B (LatB) solution, 100 μM oryzalin solution, or mock solution (0.5X MS + DMSO) two hours before inoculation with *R*. *solanacearum* K60 or *R*. *solanacearum* Δ*ripU*^*K60*^. (A) *R*. *solanacearum* K60 and Δ*ripU* ^*K60*^ colonization with LatB or mock pre-treatment. (B) *R*. *solanacearum* K60 and Δ*ripU* colonization with oryzalin or mock pre-treatment. Three independent experiments were performed each with 6 samples per treatment per genotype; all data are shown. Each dot represents a separate plant (n = 18). Wilcoxon Test: * = p<0.05, ** = p<0.01, *** = p < .001. Error bars = standard deviation.

Our data indicated that RipU^K60^ is required for the full virulence and pathogenicity of *R*. *solanacearum* K60, and that this effector associates with both actin and microtubules. In addition to these findings, a meta-analysis of three RNAseq datasets from resistant and susceptible tomatoes infected with *Ralstonia* revealed that genes involved in cytoskeletal organization were enriched among downregulated genes in susceptible tomatoes, but not in resistant plants [53] (S7 Fig). Further, pharmacological disruption of the actin or microtubule cytoskeleton can increase bacterial colonization in Arabidopsis–*P*. *syringae* interactions [29,30], and disruption of microtubule organization with oryzalin promotes colonization of *R*. *pseudosolanacearum* strain GMI100 in Arabidopsis [54]. Thus, we hypothesized that chemical disruption of the cytoskeleton would promote colonization of *R*. *solanacearum* K60 and the *ΔripU*^*K60*^ mutant. To test this, we treated roots of tomato seedlings with pharmacological inhibitors that disrupt either microtubules or actin and subsequently inoculated with *R*. *solanacearum* K60 or *ΔripU*^*K60*^. Latrunculin B (LatB) inhibits actin polymerization by binding to monomeric actin and preventing its assembly onto filament ends [55]. Oryzalin promotes the depolymerization of microtubules [56]. Prior to *R*. *solanacearum* K60 inoculation, wilt-resistant and susceptible tomato seedling roots were pre-treated with 10 μM LatB, 100 μM oryzalin, or mock treatment solution (DMSO) for two hours. Roots were then inoculated with *R*. *solanacearum* K60 or water. Cytoskeletal disruption improved colonization of both *R*. *solanacearum* and *ΔripU*^*K60*^. Wilt-resistant Hawaii7996 (H7996) and wilt-susceptible Moneymaker (MM) plants pre-treated with LatB showed significantly increased *R*. *solanacearum* K60 colonization at 24, 48, and 72 hpi when compared to roots pre-treated with mock solution (Fig 5A). LatB treatment significantly increased colonization of *ΔripU*^*K60*^ in both H7996 and MM tomatoes at 24 and 72 hpi. Colonization of *ΔripU*^*K60*^ increased with oryzalin treatment at 48 hpi but was not significantly different from mock (Fig 5A). Similar to LatB, wilt-resistant H7996 pre-treated with oryzalin showed significantly increased *R*. *solanacearum* K60 and *ΔripU*^*K60*^ colonization at 24, 48, and 72 hpi compared to roots pre-treated with mock solution (Fig 5B). In wilt-susceptible MM pre-treated with oryzalin, colonization was significantly increased at 48 and 72 hpi for *R*. *solanacearum* K60 and at 24 and 48 hpi for *ΔripU*^*K60*^ (Fig 5B).

We found no statistical difference in colonization level between tomato roots inoculated with *R*. *solanacearum* K60 and those treated with a pharmacological cytoskeletal disruptor and subsequently inoculated with *ΔripU*^*K60*^ (Fig 5A and 5B). This finding was consistent across all three timepoints and both tomato genotypes except for MM 48 hpi (P = 0.0498 for latB treatment), and H7996 48 hpi, in which oryzalin promoted significantly higher colonization by the *ΔripU*^*K60*^ mutant compared to wild type K60.

Together, these data show that pharmacological disruption of the actin or microtubule cytoskeleton promoted *R*. *solanacearum* K60 colonization in both wilt-resistant H7996 and wilt-susceptible MM roots.

### RipU^K60^ alters cytoskeleton organization

**Fig 6:**
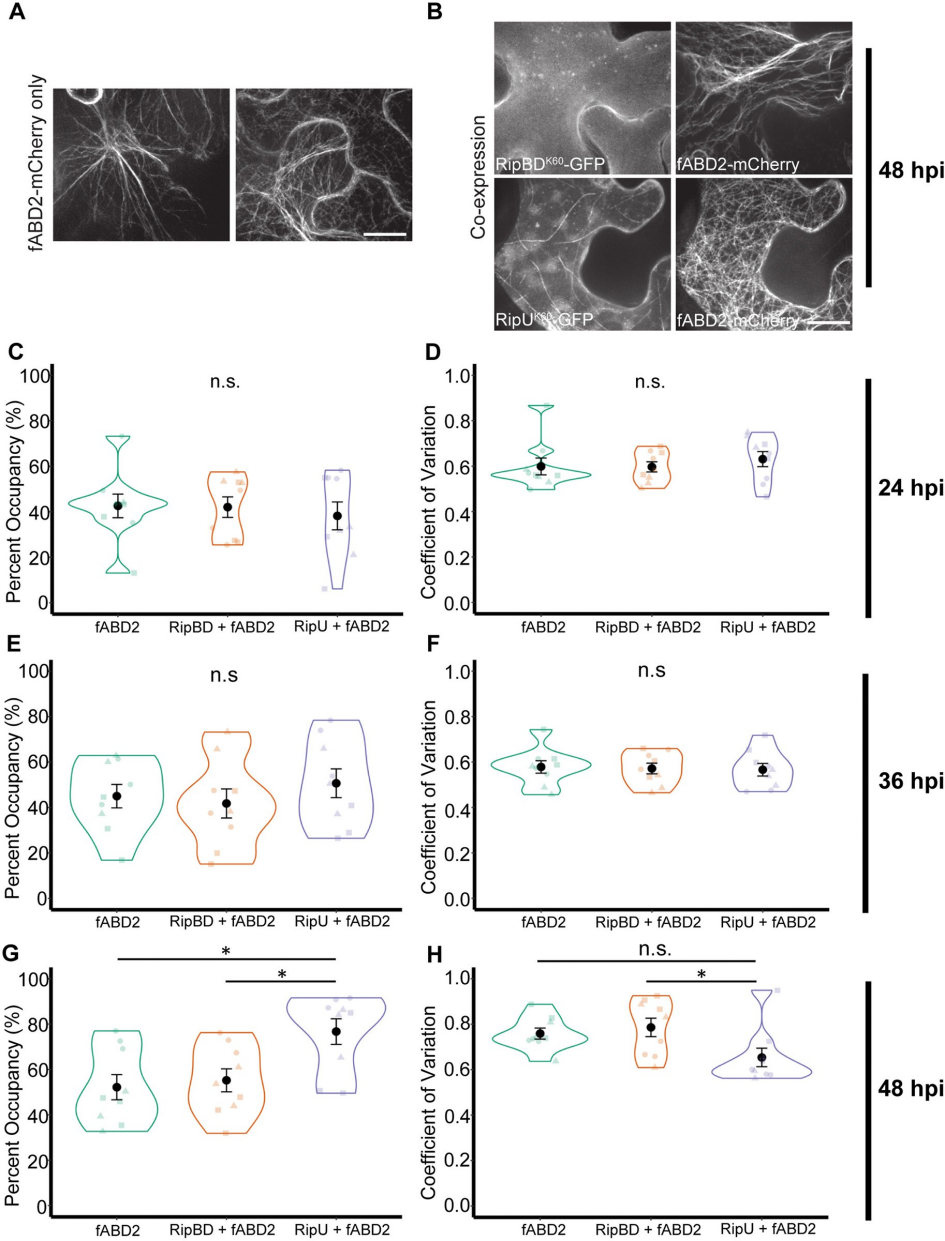
RipU^K60^ impacts actin cytoskeleton organization at 48 hours post infiltration. (A, B). Actin marker fABD2-mCherry transiently expressed in *N*. *benthamiana* leaves alone (two different cells are shown) or (B) co-expressed with either RipU^K60^ or RipBD^K60^. Representative images were taken at 48 hpi. (C,E,G) Actin filament density (Percent Occupancy) and (D, F, H) extent of actin bundling (Coefficient of Variation) were quantified at 24 (C,D), 36 (E,F) and 48 hpi (G,H). RipU^K60^ did not influence actin filament density (C, E) or bundling (D, F) at 24 (C, D) or 36 (E, F) hpi. (G,H) RipU^K60^ significantly increased actin filament density (G), and decreased actin bundling (H) compared to controls at 48 hpi. Five to fifteen cells were measured in each infiltration site and the values were averaged as one biological sample (n). Three biological samples were quantified in each of three independent experiments. Each independent experiment is depicted as different shapes within each treatment. ANOVA and Tukey test; * P < 0.05. Scale bar = 20 μm.

**Fig 7:**
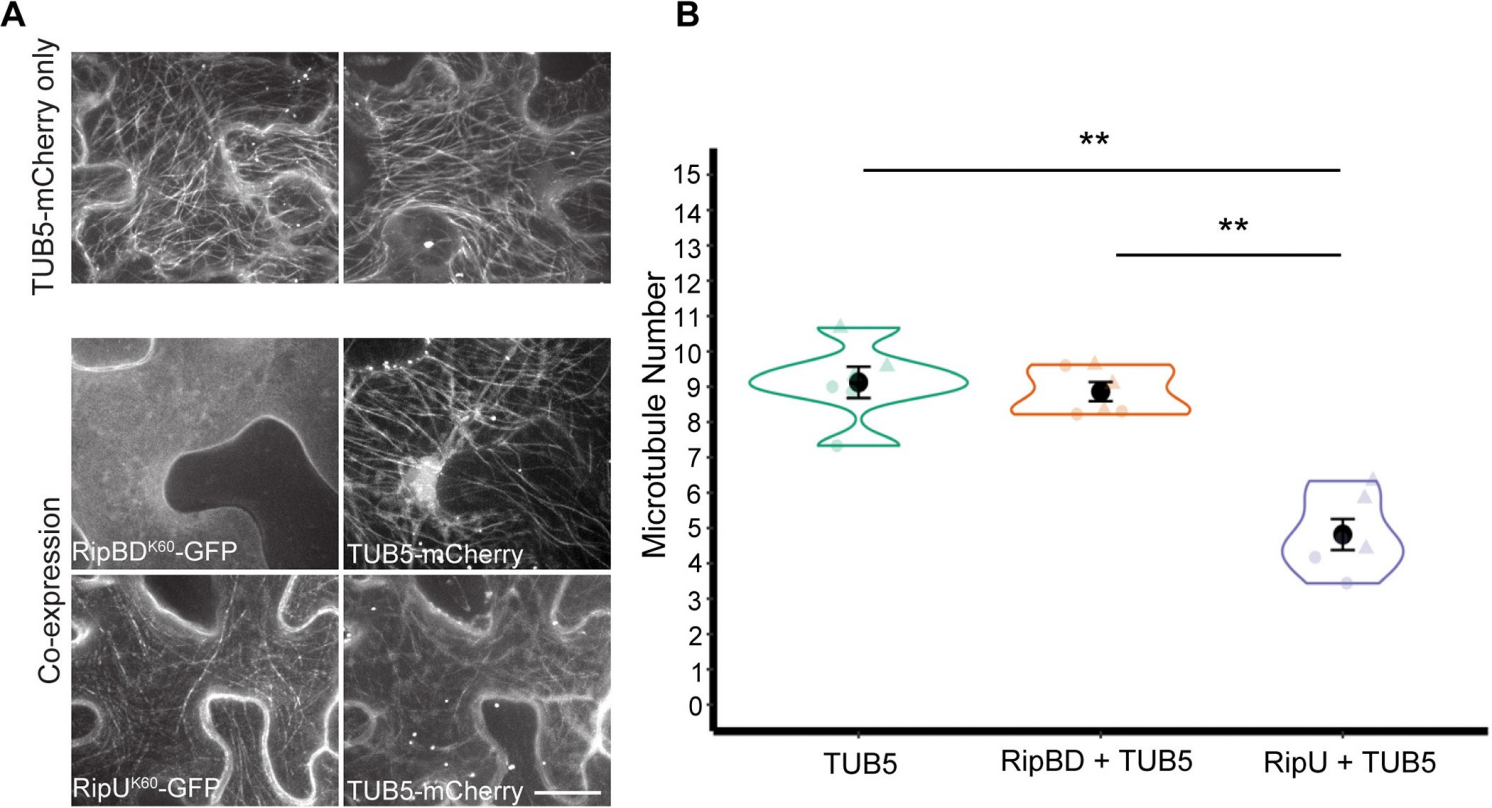
RipU^K60^ decreases microtubule abundance at 48 hours post infiltration. (A) Microtubule marker TUB5-mCherry was transiently expressed in *N*. *benthamiana* leaves alone (top, two different cells are shown) or co-expressed with either RipU^K60^ or RipBD^K60^ (bottom). Representative images are shown. (B) Heterologous expression of RipU^K60^ significantly decreased microtubule number compared to controls at 48 hpi in *N*. *benthamiana* epidermal leaves. Microtubules in five to fifteen cells were quantified in each infiltration site as described in materials and methods. Values were averaged as one biological sample (n). Three biological samples were quantified in each of two independent experiments. Each independent experiment is depicted as different shapes within each treatment. Wilcoxon rank sum test; * P < 0.05, **P < 0.01. Scale bar = 20 μm.

Given that disruption of the cytoskeleton influences the ability of *R*. *solanacearum* K60 to colonize tomato roots, and that RipU^K60^ associates with the cytoskeleton, we hypothesized that RipU^K60^ alters cytoskeleton organization. To investigate this hypothesis, we quantified actin and microtubule organization during transient expression of RipU^K60^ [19] (Figs 6 and 7). *N*. *benthamiana* leaves were co-infiltrated with *A*. *tumefaciens* strains transformed with either fABD2-mCherry or TUB5-mCherry, RipU^K60^-GFP or RipBD^K60^-GFP. Z-series of 31 optical sections were collected from epidermal cells at three different timepoints with SDCM. These z-series were processed into maximum intensity projections and used for image quantification. Cortical actin array organization was quantitatively assessed from images with two parameters: ‘Percentage occupancy’ to quantify actin filament density and ‘Coefficient of Variation (CV)’ to describe the extent of filament bundling [57,58]. Transient expression of RipU^K60^-GFP in *N*. *benthamiana* epidermal cells (Fig 6A and 6B) did not significantly influence actin filament density at 24 hpi (Fig 6C; ANOVA, p-value = 0.82) or 36 hpi (Fig 6E; ANOVA, p-value = 0.58), compared to control treatments. Similarly, at both 24 and 36 hpi, RipU^K60^ did not significantly influence filament bundling compared to controls (Fig 6D and 6F, respectively; ANOVA, 24 hpi; p-value = 0.69, 36 hpi; p-value = 0.95). However, at 48 hpi RipU^K60^ significantly increased actin filament density (Fig 6G; Tukey test, p-value for RipU-fABD2 = 0.01, p-value for RipU-RipBD = 0.026). As with our co-localization analysis, we also tested the effect of the cytoplasmic and nuclear localized effector RipAY^GMI^ on actin density at 48 hpi. When compared to RipAY^GMI^, RipU^K60^ increased actin filament density, although the effect was not statistically significant (p-value = 0.08, Tukey test, S8A Fig). At 48 hpi the extent of actin bundling significantly decreased in RipU^K60^-GFP treatments compared to the RipBD^K60^-control (Fig 6H; Tukey test, p-value for RipU-RipBD = 0.04), although was not significantly different between RipU^K60^-GFP treatment and fABD2 or RipAY^GMI^-GFP.

In contrast to our findings with the actin cytoskeleton, *N*. *benthamiana* leaves transiently expressing RipU^K60^-GFP had significantly decreased microtubule abundance at 48 hpi compared to the plasma membrane-localized effector RipBD-GFP (Fig 7A and 7B; Wilcoxon rank sum test, p-value = 0.003) and cytoplasmic and nuclear localized effector RipAY (p-value = 0.026; S8B Fig). Collectively, our results demonstrate that heterologous expression of RipU^K60^ in *N*. *benthamiana* epidermal cells leads to increased actin filament density as well as decreased microtubule numbers.

## Discussion

Here we show that a T3E protein from *R*. *solanacearum* K60 prominently colocalizes with microtubules, physically associates with actin and tubulin, is required for bacterial virulence and colonization, and alters cytoskeleton organization in heterologous expression systems. Quantitative analysis of RipU co-localization with fABD2 showed that it was not significantly different than the cytoplasmic and nuclear effector RipAY when transiently expressed in *N*. *benthamiana* cells. However, RipU associated with actin in protein interaction assays, transient expression lead to increased actin density in *N*. *benthamiana* cells, and filamentous structural overlap could be observed in SDCM images. It is possible that RipU interacts with an actin associated protein or with G-actin, the monomer form of actin. We also show that an intact cytoskeleton functions in tomato immunity to *R*. *solanacearum*, because pharmacological disruption of either actin filaments or microtubules promotes *R*. *solanacearum* colonization in tomato roots. Further, we demonstrate that RipU^K60^ is required for *R*. *solanacearum* K60 proliferation within tomato root tissue. Collectively, our data suggest that *R*. *solanacearum* K60 uses the T3E RipU^K60^ to promote its virulence *in planta* via an association with components of the cytoskeleton, likely interfering with cytoskeleton dynamics. We hypothesize that the changes in cytoskeleton organization induced by RipU^K60^ inhibit host immune signaling, further enabling *R*. *solanacearum* virulence.

### Dynamic cytoskeletal rearrangements are a critical part of plant responses to microbes

Although the cytoskeleton has been previously implicated in defense against other bacterial and fungal pathogens [4,5,7,41], our results represent significant advances in understanding the contributions of the host cytoskeleton in the tomato- *R*. *solanacearum* pathosystem. Actin remodeling is a broadly conserved plant immune response to cell surface-immune triggering microbes including the bacteria *Pseudomonas syringae*, *P*. *phaseolica*, *A*. *tumefaciens*, and the fungal pathogen *Magnaporthe grisea* [29]. For example, after inoculation with *P*. *syringae* pv *tomato* DC3000 a biphasic actin remodeling response is observed in which an initial increase in actin filament abundance peaks at 6–9 hpi, followed by enhanced bundling at 24 hpi [29]. Using different *P*. *syringae* strains and mutants, the initial response was found to be part of PTI and could be recapitulated with PAMPs, whereas the later response required the *P*. *syringae* Type III Secretion System (TTSS) and effector proteins [29]. An increase in actin filament density is also observed in hypocotyl epidermal cells upon recognition of the immunogenic peptide elf26 [37,59]. Further, co-infiltration of Arabidopsis leaves with the actin polymerization inhibitor LatB and *P*. *syringae* pv *tomato* DC3000 leads to increased bacterial growth in leaves [29,30].

One of the best characterized actin remodeling events is the focal response to leaf penetration by fungi and oomycetes, in which cortical actin arrays are reorganized into a focal actin patch and radial bundles which are focused beneath the fungal contact site [32,33,35,42,60]. The reorganization of actin is accompanied by local accumulation of endoplasmic reticulum (ER) and Golgi bodies [32,42]. Actin-dependent transport enables polarized transport and secretion of cell wall and antimicrobial compounds directly to the infection site, resulting in cell wall barriers that slow pathogen invasion as well as a localized defense response [34,37,42,61–64]. In response to the powdery mildew pathogen *Blumeria graminis* f. sp. *hordei* (*Bgh*), the increased density of actin filaments at the infection site resembles an actin patch and precedes radial bundle accumulation [35]. Formation of the actin patch requires the actin nucleator proteins ARP2/3 and the class I formin AtFH1. The actin focal response is required to prevent fungal leaf penetration, as inhibiting actin rearrangements genetically or pharmacologically promotes pathogen penetration into host cells [35,65–68].

Although actin remodeling is part of innate immune signaling [29,36,37,59], the role of microtubules in innate immunity is less clear. This is partly because a range of microtubule organization changes are elicited by microbes and the types of changes depend on the genotype of both the plant and pathogen [41]. Arabidopsis susceptibility to the necrotrophic fungal pathogen *Sclerotinia sclerotiorum* was correlated with the ability of cortical microtubules to reorganize after infection [69]. Concentrations of microtubules form beneath fungal appressoria in barley (*Hordeum vulgare*) to prevent leaf penetration of *Blumeria graminis* f. sp. *hordei* [70,71] but also in flax mesophyll cells in response to an incompatible strain of the rust fungus *Melamspora lini* [72]. In contrast, microtubules reorient or depolymerize in resistant soybean cultivars in response to the oomycete *Phytophthora sojae* [73]. Chemical disruption of microtubules with oryzalin induces expression of defense genes in grapevine [74] and promotes susceptibility to virulent bacteria [31,46]. Microtubule reorganization can be important for fungal colonization, as inhibiting microtubule reorganization promotes penetration of non-host *Blumeria* in barley [66]. Unlike actin, changes in microtubule organization in Arabidopsis have not been observed in response to pathogenic bacteria or MAMPs [31,46], but have been observed in cells from other plant species such as *Vitis rupestris* (grapevine) cells [75]. While MAMP-elicited changes to microtubule dynamics remain poorly understood, changes in microtubule organization have been observed in response to other virulence factors such as microbe-produced toxins and proteins [76–78] and in response to beneficial microbes [79]. For example, high concentrations of *Verticillium dahliae* toxin (VD toxin) disrupt microtubules and reduce microtubule density in Arabidopsis [77,78]. The limited knowledge regarding the role of microtubules in immunity coupled with the complexity of these findings represent a significant knowledge gap that has yet to be addressed in cytoskeletal response to cell surface-immune signaling.

### What is the function of the RipU^K60^ induced cytoskeletal changes?

Transient expression of RipU^K60^ in *N*. *benthamiana* decreased microtubule numbers and increased actin filament density at 48 hours following agroinfiltration. By impacting cytoskeleton remodeling, RipU^K60^ may repress cytoskeletal-mediated processes required for immune signaling, including immune receptor endocytosis and secretion of anti-microbial compounds, thereby enabling *R*. *solanacearum* colonization. We also observed that RipU ^K60^ and fABD2 co-localization occurred in part around intracellular compartments. This observation appears remarkably similar to that of actin filament baskets, which have been previously described as a proliferation of actin filaments surrounding plastids [80]. These actin baskets were described to play a role in plastid movement and orientation [80], however, it currently remains unknown whether RipU^K60^ shares these functions.

Cytoskeleton disruption may also indirectly promote *R*. *solanacearum* growth by enabling *R*. *solanacearum* K60 to gain better access to the vasculature. The microtubule cytoskeleton plays an important role in secondary cell wall formation in the xylem [81,82]. Trafficking of cellulose synthase complexes (CSCs) along cortical microtubules enables placement of cellulose in xylem cell walls [83–85]. As the xylem develops and undergoes programmed cell death, cell walls thicken and lignify. Lignification is thought to play a role in preventing the penetration of the xylem by pathogens as vascular pathogens need to overcome this barrier to access the xylem [86,87]. Notably, not all areas of the xylem cell wall are lignified. *R*. *solanacearum* appears to spread among xylem cells in part through pits which form between xylem cells [88]. Local depolymerization of microtubules prevents CSCs from moving to the region, thereby inhibiting secondary cell wall thickening [85,89]. By promoting the depolymerization of microtubules, RipU^K60^ may alter secondary cell wall structure, thereby decreasing lignification and potentially increasing pit formation, thus allowing *R*. *solanacearum* entry into the vasculature.

### Elucidating the mechanisms underlying the interaction between RipU^K60^ and the cytoskeleton

We have shown that RipU^K60^ co-localizes and associates with components of the cytoskeleton. However, it is possible that such an association may be with the monomer form of actin or be indirect and mediated by interactions with cytoskeleton-associated proteins. For example, we identified the actin binding protein (ABP) Actin Depolymerizing Factor 4 in the RipU co-IP/MS results. Given that RipU^K60^ influences actin dynamics and microtubule number, RipU^K60^ may interact with components required for cytoskeleton remodeling, such as ABPs and microtubule-associated proteins (MAPs), or it may alter second messenger production (e.g. ROS, Ca^2+^, or phospholipids) that modulate cytoskeletal dynamics. Alternatively, RipU^K60^ could interfere with actin-microtubule crosstalk by associating with proteins that interact with both actin and microtubules [90]. The HopG1 effector from *P*. *syringae* triggers the actin reorganization observed during effector triggered susceptibility [43] and interacts with a kinesin, a microtubule-associated motor protein. HopG1 co-immunoprecipitates with actin when kinesin is present but not when expressed alone [43]. Although the major function of kinesins is to move vesicles directionally along microtubules, these motor proteins can also cross-link actin to microtubules [91]. A kinesin mutant has reduced susceptibility to *Pst*, suggesting that HopG1 targets kinesin to promote actin changes that enhance pathogen virulence [43]. If RipU^K60^ interferes with actin-microtubule crosstalk, this may simultaneously alter both components of the cytoskeleton, actin immune signaling, as well as downstream cell wall assembly resulting from microtubule disruption.

### RipU^K60^ is unusual among T3Es that impact the cytoskeleton

Given the central role of the cytoskeleton in immunity, it is unsurprising that microbial pathogens have evolved effector proteins that target and suppress cytoskeletal functions. Although multiple T3Es interact with the cytoskeleton, they do not alter its organization in the same way. For example, *Xanthomonas campestris* XopR directly interacts with the actin nucleating protein formin and promotes actin nucleation during early stages of infection. High concentrations of XopR inhibit nucleation and cause formin aggregation [39]. *P*. *syringae* DC3000 HopG1 induces actin bundling and decreases actin filament density in Arabidopsis cotyledons [43]. HopG1 does not impact *P*. *syringae* DC3000 growth in Arabidopsis but promotes symptom (chlorosis) development. The chlorosis induced by *P*. *syringae* DC3000 appears to be linked to changes in actin organization. Inoculating plants with DC3000 and inhibiting actin polymerization with cytochalasin D led to enhanced chlorosis, while promoting actin polymerization with jasplakinolide inhibited symptom development [43]. HopW1 from *P*. *syringae* co-immunoprecipitates with Arabidopsis Actin7 and, in contrast to RipU^K60^, reduces actin filament density when expressed in *N*. *benthamiana* or Arabidopsis at 6, 24 and 48 hpi [30]. HopW1 disrupts endocytosis during early infection in Arabidopsis [30] and may impact recycling of immune-related proteins at the cell surface.

Additional effectors target microtubules or microtubule-related proteins. HopZ1a, a T3E from. *syringae* binds and acetylates tubulin [31]. Expression of HopZ1a causes a significant decrease in the density of microtubule networks in Arabidopsis, disrupts the plant secretory pathway, and suppresses callose formation [31]. Whether HopZ1a impacts the actin cytoskeleton is not known. HopE1 interacts with MAP65-1 in a calmodulin-dependent manner [46]. Binding of HopE1 from *Pst* DC3000 to calmodulin leads to the disassociation of MAP65-1 from microtubules but does not appear to impact the organization of microtubules. AvrBsT acetylates Arabidopsis ACIP1, which positively regulates immune responses and co-localizes with microtubules. Acetylation of AtACIP1 by AvrBsT changes this co-localization and promotes the aggregation of large AtACIP1 puncta throughout the cell [47]. Whether microtubule localization is required for the immune-related function of AtACIP1 remains unclear.

To the best of our knowledge, only the T3E protein RipU^K60^ has been shown to physically associate with both actin and microtubules. However, several lines of evidence suggest that other effectors may also interfere with multiple structures of the plant cytoskeleton. Transient expression of XopL from *X*. *euvesicatoria* causes cell death in *N*. *benthamiama* and decreases microtubule number in *N*. *benthamiama* epidermal cells [45]. Co-localization of XopL with microtubules is correlated with the cell death phenotype, as XopL truncations that did not strongly co-localize with microtubules also did not cause cell death [45]. As discussed above, HopG1 alters actin organization but interacts with a microtubule motor protein. Although the impact of HopG1 expression on microtubule organization was not tested, these data suggest that interactions between actin and microtubules are important for host immune responses and suggest that proteins that interact with both actin and microtubules are possible targets for effector proteins.

### Conclusions

Effector proteins are often functionally redundant and/or work cooperatively to target common functions in host cells [1,18]. Targeting the cytoskeleton may serve not only as a platform for interfering with multiple plant functions but may provide a way for effector proteins to interact synergistically and gain additional functionality. Given the close relationship of the cortical cytoskeleton with the plasma membrane and cell wall [92], as well as the localization of many Rips to the cell periphery [19], we speculate that RipU^K60^ may function with other Rips to modulate immune processes at the cell periphery. For example, since microtubules influence cellulose alignment, one can conceive of RipU^K60^ functioning with another effector to alter cellulose synthase complex placement and cell wall structure. Alternatively, since actin promotes plasma membrane nanodomain formation [93], RipU^K60^ could function alongside other Rips to change the placement of immune receptors on the plasma membrane.

Together our data suggest that by preventing proper cytoskeleton remodeling, RipU^K60^ represses cytoskeletal-mediated processes required for immune signaling, thereby enabling *R*. *solanacearum* colonization. The discovery of a T3E that targets multiple components of the cytoskeleton underscores the importance of this proteinaceous network for immunity. The molecular mechanisms through which RipU^K60^ alters the cytoskeleton and the specific impacts of RipU^K60^-induced cytoskeletal disruptions (for example changes to PRR endocytosis) remain unknown but will be the subject of future investigation.

## Materials and Methods

### Plasmid construction

Full-length RipU^K60^ was PCR-amplified from *R*. *solanacearum* K60 genomic DNA; RipAY^GMI^ was amplified from *R*. *pseudosolanacearum* GMI1000. The resulting PCR products were cloned into either pENTR/D-TOPO or pBSDONR P1-P4 [94,95] Gateway donor plasmids using BP Clonase II (Invitrogen). We designated the resulting clones pENTR/D-TOPO-RipU^K60^ (or RipAY^GMI^) and pBSDONR (P1-P4)-RipU^K60^ or RipU^GMI^.

To generate the Green Fluorescent Protein (GFP)-tagged RipU^K60^ construct, the pENTR/D-TOPO-RipU^K60^ or RipAY^GMI^ plasmid was mixed with the Gateway-compatible destination plasmid pB7FWG2, which places the transgene under the control of the 35S promoter [96]. Plasmids were recombined by addition of LR Clonase II (Invitrogen) following manufacturer instructions. The resulting expression clone was designated pB7FWG2-RipU^K60^-GFP or RipAY^GMI^- GFP.

The pB7FWG2:RipU^K60^ or RipAY^GMI^ construct was sequence-verified and subsequently mobilized into *Agrobacterium tumefaciens* GV3101 (pMP90).

### Agar plate-based plant growth conditions

Resistant tomato accession Hawaii7996 (H7996; *Solanum lycopersicum*), susceptible MoneyMaker (MM; *S*. *lycopersicum*) and susceptible L390 (*S*. *lycopersicum* var. *cerasiforme*) seeds were surface disinfested with a 50% bleach solution for 5 minutes, washed with water and stratified at 4°C overnight. Seeds were then planted on water-agar and grown at 28°C to 30°C in a growth chamber at 16h:8h day/night cycle.

### Soil-based plant growth conditions

Sterilized and stratified tomato seeds were planted in Pro-Mix PGX Propagation soil (Premier Tech Horticulture) in 3603 pots containing 25-27g of soil. Plants were grown under 16h:8h day/night cycle, at 28°C to 30°C in a growth chamber for 15 days prior to inoculation with *Ralstonia*. At 10 days post planting, tomato seedlings were fertilized with Peters Fertilizer (Hummert International, USA).

*Nicotiana benthamiana* seeds were sown in pots containing Pro-Mix PGX Propagation soil (Premier Tech Horticulture) and grown for 4 to 5 weeks in a growth chamber at 60% relative humidity in a 16-h-:8-h day/night cycle at 22°C to 23°C.

### Secretion assay

The secretion assays were performed as in [48]. The pAM5 plasmid (a gift from Stephane Genin) was electroporated into both *Ralstonia pseudosolanacearum* strain GMI1000 *(Rp*^GMI^) and *ΔhrcC*^*GMI*^ mutant strains carrying RipU^K60^-3HA fusion. The *ΔhrcC*^*GMI*^ mutant strain lacks a functional type III secretion system and is unable to secrete T3Es. The plasmid pAM5 increases *HrpB* expression and allows for better detection of the effector in the culture supernatant. pAM5 was electroporated into transformed *Rp*^*GMI*^ or the *ΔhrcC*^*GMI*^ mutant. Transformed *Rp*^*GMI*^ and the *ΔhrcC*^*GMI*^ mutant were cultured overnight at 28°C and pellets were resuspended in secretion media. After an 8h incubation at 28°C, samples were diluted with the secretion medium to ensure equal optical density (O.D.) readings (O.D._600_ 0.5–0.8). The supernatant and the pellet were then separated via centrifugation. Bacterial pellets were resuspended in sterile water and stored at -20°C. Supernatant samples were filtered, 1 mL of cold 25% trichloroacetic acid was added and incubated overnight at 4°C. The pellets of the supernatant were washed with 1 mL acetone 90%, dried, and stored at -20°C.

### Agrobacterium-mediated transient expression in Nicotiana

The pB7FWG2-RipU^K60^-GFP construct was transformed into *A*. *tumefaciens* GV3101 (pMP90) and streaked onto Luria-Bertani (LB) media supplemented with 25 μg/ml gentamicin sulfate and 100 μg/ml spectinomycin. Cultures were prepared in liquid LB, with the appropriate antibiotics, and grown overnight at 30°C. Following overnight incubation, cells were pelleted by centrifugation at 3,000 x g for 3 minutes at room temperature and resuspended in 10 mM MgCl_2_. Bacterial suspensions were diluted to an optical density at 600 nm (OD_600_) of 0.5, incubated in 150 μM acetosyringone for 3–4 hours at room temperature, and infiltrated into four-week *Nicotiana benthamiana*. For co-infiltration experiments, *A*. *tumefaciens* strain GV3101 harboring 35S::fABD2-mCherry or UBQ10::TUB5-mCherry constructs were co-infiltrated into *N*. *benthamiana* with RipU^K60^-GFP or RipBD^K60^-GFP. Infiltrated leaves were imaged at 24 and 48 hpi.

### Cytoskeleton imaging and quantitative image analysis

*N*. *benthamiana* leaf epidermal cells co-expressing cytoskeletal markers and *R*. *solanacearum* T3Es were imaged by spinning disc confocal microscopy (SDCM) with an Olympus IX-83 inverted microscope equipped with a spinning disc confocal head (Yokagawa CSU-X1-A1; Hamamatsu Photonics, Hamamatsu, Japan) and an Andor iXon Ultra 897BV EMCCD camera (Andor Technology, Concord, MA, USA). Images were collected with an Olympus 60x oil objective (1.40 NA UPlanSApo; Olympus) using MetaMorph version 7.10.5 software. GFP and mCherry fluorescence were excited with 488-nm and 561-nm lasers and emission collected through 525/30-nm and 607/36-nm filters, respectively. Confocal z-series were collected at 0.5 μm step size for a total of 30 steps. For GFP and mCherry double-channel imaging, cells expressing single markers were checked to make sure no fluorescence bleed-through was detected in each channel.

All image processing and analysis were performed in ImageJ or FIJI [97]. Epidermal cell z-series were converted into single images by maximum intensity projection of either the entire stack or the uppermost two slices (for experiments with RipAY in S5 Fig and S8 Fig) before quantitative analysis. For colocalization analysis of RipU^K60^-GFP with actin filaments or microtubules, intracellular regions co-expressing both markers were cropped in both channels and used as Region of Interests (ROIs) for Pearson’s correlation coefficient analysis. The analysis was performed with the ImageJ plug-in JaCoP [98]. Cells co-expressing RipBD^K60^-GFP and actin or microtubule markers were analyzed in the same experiments as control for random colocalization.

Actin density was analyzed as percentage of occupancy as previously described [29]. Actin extent of bundling was analyzed by quantifying the coefficient of variation (CV) of intensity values with an ImageJ macro developed by [58]. Microtubule abundance was estimated by counting microtubule numbers along a 10 μm line drawn vertically to the orientation of the most microtubules in a cell as previously described [99]. For all quantitative analysis, 5–15 cells were measured at each infiltration site and at least three infiltration sites were measured in each experimental replicate.

### *R. solanacearum* recombinant DNA techniques

A clean deletion mutant Δ*ripU*^*K60*^ was created using *sacB* counter-selection with the vector pK18mobsacB as previously described [100]. Briefly, the upstream (646 bp) and downstream (536 bp) regions of *ripU* were amplified from *R*. *solanacearum K60* gDNA using Q5 high fidelity DNA polymerase (New England Biolabs, Ipswich, MA, USA) with the primers *ripU* up F/R and *ripU* dw F/R (S2 Table). Upstream and downstream fragments were fused and cloned at the HindIII site into pK18mobsacB by Gibson Assembly (New England Biolabs, Ipswich, MA, USA) following the manufacturer’s recommendations. pK18mobsacBΔ*ripU* construct was inserted into K60 using electroporation as previously described [101].

The first genomic recombination event was selected on CPG + Km. The second recombination event was screened for sucrose and Km sensitivity on CPG + 10% sucrose. Successful deletion of *ripU* (Δ659 bp) was confirmed using PCR with primers *ripU*flank F/R (S2 Table). Genomic DNA was isolated using Genomic DNA Buffer Set with Genomic-tip 20/G (Qiagen, Hilden, Germany).

To construct the complementation vector, the gene region including the native promoter (405 bp upstream) and terminator (269 bp downstream) was polymerase chain reaction (PCR)–amplified from *K60 gDNA* and cloned via Gibson Assembly (New England Biolabs, Ipswich, MA, USA) at the HindIII site of pUC18miniTn7Gm to create pUC18miniTn7Gm::*ripU*^*K60*^, following the manufacturer’s protocol. K60Δ*ripU* was transformed with pUC18miniTn7Gm::*ripU*^*K60*^ and pTNS3 to promote transposition and single gene insertion.

### Soil Drench Inoculation

Wild type *R*. *solanacearum* K60, the *ΔripU* ^*K60*^ mutant, and the *ΔripU* ^*K60*^::*RipU* ^*K60*^ complementation mutant were grown for two days on Casamino Peptone Agar (CPG) containing 1% triphenyl tetrazolium chloride (TZC) at 28°C. Bacteria were harvested and resuspended in sterile water to 10^8^ CFU/mL. Tomato plants were inoculated at the three-leaf stage by applying inoculum (wild-type *R*. *solanacearum* K60, *ΔripU* ^*K60*^, and *ΔripU* ^*K60*^::*RipU* ^*K60*^) or water (mock treatment) directly to the soil using a serological pipette (1mL of inoculum/1 mg of soil) as described in Meline et al. 2023 (53).

### Root colonization and disease index

To measure *R*. *solanacearum* K60 colonization, roots from inoculated tomato plants were harvested at 24, 48 and 72 hours post inoculation. Within treatments, harvested roots were pooled in groups of three and weighed after the removal of residual soil and water. The surface of these pooled roots was then sterilized. Root samples were ground with a mortar and pestle and resuspended with 1 mL of sterile water. This root tissue slurry was used to plate serial dilutions on CPG + 1% TZC to measure the colony forming units (CFUs) of *R*. *solanacearum* K60 present per gram of root tissue. These dilution plates were incubated at 28°C for 48 hours. To determine the pathogen titer, colonies were counted on the dilution plates and set relative to the mass of the original root tissue. Experiments were repeated three independent times.

Inoculated tomato plants were scored daily for wilting severity to assess bacterial wilt disease index. Wilting symptoms were quantified on a scale from 0 to 4 (0 = no leaves with observable wilting, 4 = 100% of leaves wilting). Using these raw wilt scores, disease index was calculated using the following equation (*DI* = Disease Index, *n_w_* = number of leaves wilted, *n* = total number of leaves).


$$DI=\frac{n_{w}}{n}$$ (4)

Experiments were replicated three independent times. Area Under the Disease Progress Curve (AUDPC) was assessed with the formula A = $\sum_{i=1}^{N_{i-1}}(\frac{y_{i}+y_{i+1}}{2})(t_{i+1}-t_{i})$, where y = disease severity on day i, and t = time after inoculation.

### Cytoskeleton disruption and colonization assays

Tomato seedlings were grown on water agar and treated with either 10 μM latrunculin B (LatB) solution, 100 μM oryzalin solution, or mock treatment solution (DMSO) two hours prior to inoculation with *R*. *solanacearum*. *R*. *solanacearum* was inoculated by pipetting 200 μl inoculum onto the entire root. Treatments were allowed to dry in a sterile hood. *R*. *solanacearum* K60, *ΔripU*^*K60*^ inoculum (1x10^5^ CFU/ml) or a mock treatment (water) was then applied to root tips. There were six treatments for these experiments; *R*. *solanacearum* K60, *R*. *solanacearum* K60 + LatB, *R*. *solanacearum* K60 + oryzalin, *ΔripU*^*K60*^, *ΔripU*^K60^ + LatB and *ΔripU*^K60^ + oryzalin. Colonization assays were performed on inoculated roots. Colonization experiments were performed in three independent experiments. Data were not normally distributed. Statistical analysis was performed in R (version 3.6.1).

### Co-immunoprecipitation assay

Co-immunoprecipitation (co-IP) were conducted on protein extracted from *Nicotiana benthamiana* leaves expressing the green fluorescent protein (GFP) epitope tagged proteins as described previously [102] with slight modifications. Briefly, three leaves were harvested and pooled at 48hpi and preserved with liquid nitrogen. Leaf tissues were ground in 1mL of ice cold IP buffer (150 mM NaCl, 50 mM Tris [pH 7.5], 10% glycerol, 1 mM dithiothreitol, 1 mM EDTA, 1% Nonidet P-40, 0.1% Triton X-100, 1% plant protease inhibitor cocktail, and 1% 2,2’-dipyridyl disulfide) using a cold ceramic mortar pestle and were centrifuged at 10,000 x g for 15 minutes at 4°C. The supernatant was incubated with 10μL of green fluorescent protein (GFP)-Trap A (Chromotek) bead slurry for 4 hours at 4°C with constant slow rotation followed by washing the bead slurry 5 times with 500μL IP wash buffer at 1,000 x g for 1 minute at 4°C. The beads were resuspended in IP buffer and combined with 4x Laemmli buffer (BioRad) supplemented with 10% β-mercaptoethanol and the mixtures were boiled at 95°C for 10 min. 20 μl of input and 5 μl of IP samples were loaded and protein samples were separated on 4–20% Tris-glycine polyacrylamide gels (Bio-Rad) at 170 V for 1 hour in 1X Tris/glycine/SDS running buffer. Total proteins were transferred to nitrocellulose membranes (GE Water and Process Technologies) at 100 V for one hour. Membranes were incubated with blocking buffer (1X Tris-buffered saline (50 mM Tris-HCl, 150 mM NaCl [pH 6.8]) solution containing 0.1% Tween20 (TBST) containing 5% Difco skim milk) for 1 hour at room temperature with gentle shaking. Proteins were subsequently detected with horseradish peroxidase (HRP)-conjugated anti-GFP antibody (1:5,000) (Miltenyi Biotec #130-091-833), anti-plant actin mouse monoclonal antibody (3T3)-HRP (Abbkine # A01050HRP), alpha Tubulin monoclonal primary antibody (YL1/2) (1:10,000; Thermo Scientific MA1-80017) and goat anti-rat IgG secondary antibody (1:10,000; Thermo Scientific PA1-84709) in blocking buffer. Following antibody incubation, membranes were washed at least three times for 10 minutes with 1x TBST solution followed by 5 minutes incubation at room temperature with either Clarity Western ECL (BioRad). Immunoblots were developed using an Amersham ImageQuant 500 CCD imaging system (Cytiva). The experiment was repeated three times with similar results.

### Mass-spectrometry analysis

The immune precipitated samples were subjected to mass spectrometric analysis following the standard bottom-up proteomics protocol with modifications as described in [103,104]. ~1ug desalted peptides were injected into Dionex ultimate 3000 RSLC nano LC-system, coupled to an Orbitrap Q-exactive^HF^ instrument. The mass spectrometry settings were followed as described in [105]. Briefly, peptides were first loaded into the C18 trap column (trap cartridge) (Thermo Fisher Scientific, Waltham, MA, USA) and then separated using an analytical reverse phase 1.7 μm 120 Å IonOptics Aurora Ultimate C18 column (75 μm x, 25cm). The column was maintained at 45°C, mobile phase solvent A was 0.1% FA in water, and solvent B was 0.1% FA in 80% ACN. The loading buffer was 0.1% FA in 3% ACN. Peptides were loaded into the trap column for 5 min at 5 μl^-1^, then separated with an analytical column at a flow rate of 400 nl min^-1^ for 112 mins. The columns were conditioned for 130 mins. The mass spectrometer was operated in positive ion and standard data-dependent acquisition (DDA) mode. The spray voltage was set at 2.5 kV, the capillary temperature was 320°C and the S-lens RF was set at 50. The resolution of Orbitrap mass analyzer was set to 120,000 and 15,000 at 200 m/z for MS1 and MS2, respectively, with a maximum injection time of 100 ms for MS1 and 20 ms for MS2. The full scan MS1 spectra were collected in the mass range of 350–1600 m/z, and the MS2 first fixed mass was 20 ms. The automatic gain control (ACG) target was set to 3 × 10^6^ for MS1 and 1 × 10^5^ for MS2. The fragmentation of precursor ions was accomplished by higher energy Ctrap collision dissociation (HCD) at a normalized collision energy setting of 27% and an isolation window of 1.2 m/z. The DDA settings were for a minimum intensity threshold of 5 × 10^4^ and a minimum AGC target of 1 × 10^3^. The dynamic exclusion was set at 50 s and accepted charge states were selected from 2 to 7 with 2 as a default charge. The data was collected in centroid version.

### Proteomic raw data analysis

Proteomics raw data were processed using the Proteome Discoverer (version 3.1, Thermofisher Scientific) software using tobacco proteome (Uniprot), including bait sequence. A standard search workflow for the hybrid and LTQ instruments was used to search. An in-house list of common contaminants was added to the search. Sequest HT tool was used to assign the peptides, allowing a maximum of two missed tryptic cleavages, a minimum peptide length of six, a precursor mass tolerance of 10 ppm, and a fragment mass tolerance of 0.02 Da. Carbamidomethylation of cysteines and oxidation of methionine were specified as static and dynamic modifications, respectively. A false discovery rate of high-confidence validated peptide spectral matches was used for the downstream analysis. Label-free quantification based on MS1 precursor ion intensity was performed in Proteome Discoverer with a minimum quan value threshold set to 0.001 for unique peptides; the ‘3 Top N’ peptides were used for area calculation. The normalized protein abundances were calculated among the measured samples, and values were Log transformed and imputed following a normal distribution pattern. R-studio, Perseus and Microsoft excel were used to generate the graphs and compute the data. Adobe Illustrator and Microsoft PowerPoint was used to present graphical visuals.

### Yeast two-hybrid assays

RipU and *Sl*Actin (*Solyc10g086460*) were cloned using Gateway technology into pDEST32 (‘bait vector’ with the DNA binding domain) or pDEST22 vector (‘prey’ vector with Gal4AD) in the Proquest Yeast-Hybrid System (Invitrogen). After sequence confirmation, constructs were transformed into yeast MaV203 cells (Invitrogen) following the manufacturer’s instructions. Single colonies were plated onto yeast minimal media (SD base) with appropriate dropout supplements (Takara Bio) and grown at 28°C.

### Statistical analysis

Statistics were performed in R. studio v 3.6.1 or JMP Pro 17. Data were tested for normality. A parametric or non-parametric test was chosen depending on whether the data met the assumptions for a given statistical test. The number of experimental replicates and biological samples used for each experiment is stated in each figure legend. Data used to generate graphs are in S3 Table.

## Supporting information

S1 Fig RipU is secreted through the type III secretion system. Immunoblots with HA-tagged RipU^K60^ (RipU^K60^:HA = ~33kDa) detected in (A) both the supernatant and pellet of *R*. *pseudosolanacearum* GMI1000 and (B) only in the pellet of the *ΔhrcC* mutant.
(PDF)

S2 Fig Transient expression of RipU in *N*. *benthamiana*. 35S:RipU^K60^:GFP was transiently expressed in *N*. *benthamiana* leaves using agroinfiltration. **(A)** Tissue was collected at 24 and 48 hpi and immunoblot detection performed with anti-GFP antibody. **(B)** At 96 hpi no visible differences in leaves were observed between leaves transiently expressing 35S:RipU^K60^:GFP and those expressing 35S:GFP or empty vector. Dotted lines indicate area of infiltration.
(PDF)

S3 Fig RipU^K60^-GFP and fABD2-mCherry surround intracellular components. Spinning disk confocal images at 24 (top) and 36 (bottom) hpi. The actin marker and RipU appear to co-localize surrounding an unidentified intracellular component. Arrows point to compartments.
(PDF)

S4 Fig Spinning disk maximum projection image of RipAY^GMI^-GFP. (PDF)

S5 Fig Co-localization analysis of RipU and the cytoplasmic and nuclear localized effector RipAY with the cytoskeleton 48 hpi in transient expression assays in *N*. *benthamiana* leaves. Spinning disk confocal images of RipAY^GMI^:GFP and RipU^K60^:GFP co-infiltrated with either (A) actin reporter fABD2-mCherry or (B) microtubule reporter mTUB5-mCherry. Images were captured 48 hpi after agroinfiltration. Five to fifteen cells were measured at each infiltration site and the values were averaged as one biological sample (n). Three biological samples were quantified in each of two independent experiments. Each independent experiment is depicted as a different shape within each treatment. (C) Pearson’s colocalization analysis of RipU, RipAY and RipBD with fABD2. RipBD images are not shown but were performed as part of the same experiment. (D) Pearson’s colocalization analysis of RipU, RipAY and RipBD with mCherry. RipBD images are not shown but were performed as part of the same experiment. Letters indicate significance with a Tukey’s test after one-way ANOVA.
(PDF)

S6 Fig Principal component analysis (PCA) between the three Co-IP independent experiments between 35S:GFP and 35S:RipU:GFP. (PDF)

S7 Fig Cytoskeleton-related Gene Ontology (GO) categories are enriched among down-regulated genes in a meta-analysis of *Ralstonia*-infected tomatoes. (A) GO biological process categories related to the cytoskeleton that are enriched among downregulated genes in susceptible tomatoes in Meline et al. 2023 (53). (B) Genes in the categories in (A) and their closest Arabidopsis homolog identified from Phytozome 13.
(PDF)

S8 Fig Actin and microtubule organization 48 hpi after transient expression of RipAY, RipBD, RipU and fABD2 in *N*. *benthamiana* epidermal leaf cells. (A) Actin density (percent occupancy). (B) Microtubule number. Five to fifteen cells were measured at each infiltration site and the values were averaged as one biological sample (n). Three biological samples were quantified in each of two independent experiments. Each independent experiment is depicted as a different shape within each treatment. Letters indicate significance with a Tukey’s test after ANOVA.
(PDF)

S1 Table Raw and processed data for Co-IP/MS analysis. LC-MS/MS data from the three independent Co-IP experiment were combined and analyzed together. Only 35S: GFP and 35S:RipU:GFP protein eluant samples were used for downstream analysis.
(XLSX)

S2 Table Primers for *R*. *solanacearum* Recombinant DNA techniques. (XLSX)

S3 Table Data used to generate all graphs. (XLSX)
