# Supplementary material for: A Ralstonia solanacearum type III effector alters the actin and microtubule cytoskeleton to promote bacterial virulence in plants
Source: PLoS Pathog. 2024 Dec 26;20(12):e1012814. doi: 10.1371/journal.ppat.1012814 (PMC11723619; doi:10.1371/journal.ppat.1012814)
Supplement: S1 Fig — Immunoblots with HA-tagged RipUK60 (RipUK60:HA = ~33kDa) detected in (A) both the supernatant and pellet of R. pseudosolanacearum GMI1000 and (B) only in the pellet of the ΔhrcC mutant. (PDF) [file ppat.1012814.s001.pdf]

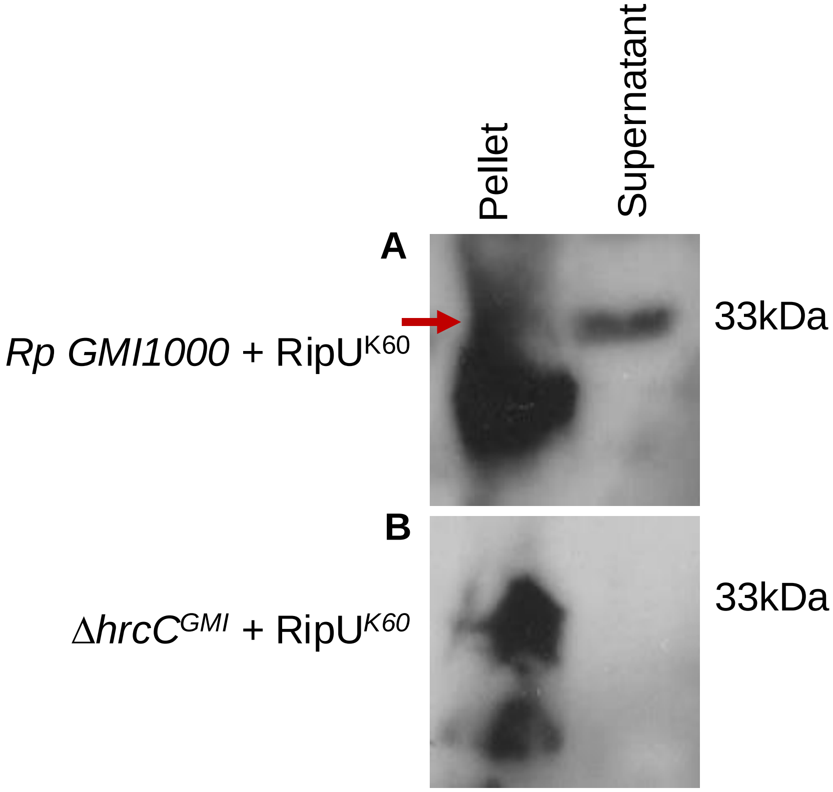


**Supporting Fig 1. RipU is secreted through the type III secretion system.** Immunoblots with HA-tagged $RipU^{K60}$ ($RipU^{K60}$:HA = ~ 33kDa) detected in (A) both the supernatant and pellet of *R. pseudosolanacearum* GMI1000 and (B) only in the pellet of the Δ*hrcC* mutant.
