# Supplementary material for: A Ralstonia solanacearum type III effector alters the actin and microtubule cytoskeleton to promote bacterial virulence in plants
Source: PLoS Pathog. 2024 Dec 26;20(12):e1012814. doi: 10.1371/journal.ppat.1012814 (PMC11723619; doi:10.1371/journal.ppat.1012814)
Supplement: S2 Fig — 35S:RipUK60:GFP was transiently expressed in N. benthamiana leaves using agroinfiltration. (A) Tissue was collected at 24 and 48 hpi and immunoblot detection performed with anti-GFP antibody. (B) At 96 hpi no visible differences in leaves were observed between leaves transiently expressing 35S:RipUK60:GFP and those expressing 35S:GFP or empty vector. Dotted lines indicate area of infiltration. (PDF) [file ppat.1012814.s002.pdf]

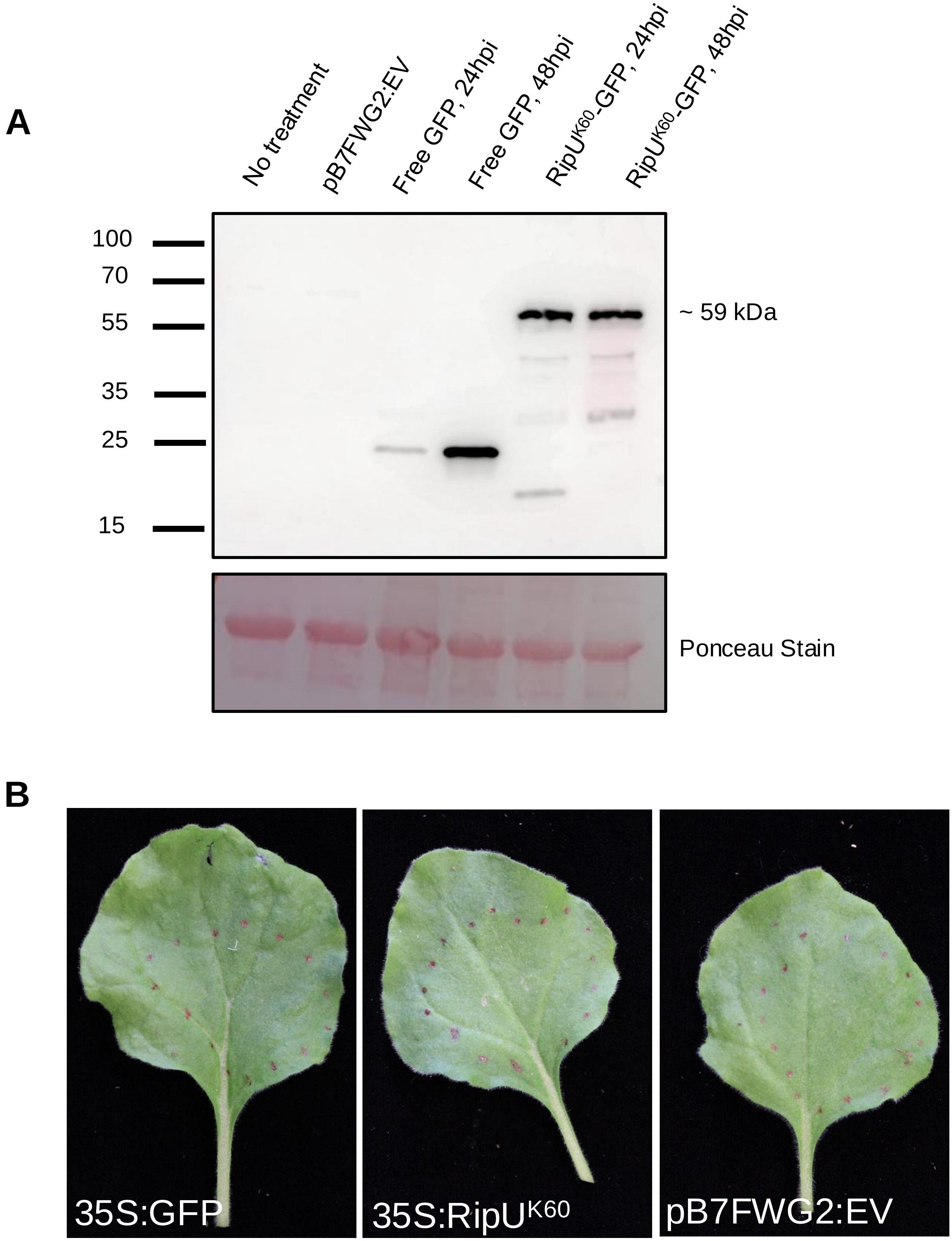

Supporting Fig 2: Transient expression of RipU in *N. benthamiana*.
35S:RipU^{K60}:GFP was transiently expressed in *N. benthamiana* leaves using agroinfiltration. **(A)** Tissue was collected at 24 and 48 hpi and immunoblot detection performed with anti-GFP antibody. **(B)** At 96 hpi no visible differences in leaves were observed between leaves transiently expressing 35S:RipU^{K60}:GFP and those expressing 35S:GFP or empty vector (EV). Dotted line indicates area of infiltration.
