# Supplementary material for: A Ralstonia solanacearum type III effector alters the actin and microtubule cytoskeleton to promote bacterial virulence in plants
Source: PLoS Pathog. 2024 Dec 26;20(12):e1012814. doi: 10.1371/journal.ppat.1012814 (PMC11723619; doi:10.1371/journal.ppat.1012814)
Supplement: S3 Fig — Spinning disk confocal images at 24 (top) and 36 (bottom) hpi. The actin marker and RipU appear to co-localize surrounding an unidentified intracellular component. Arrows point to compartments. (PDF) [file ppat.1012814.s003.pdf]

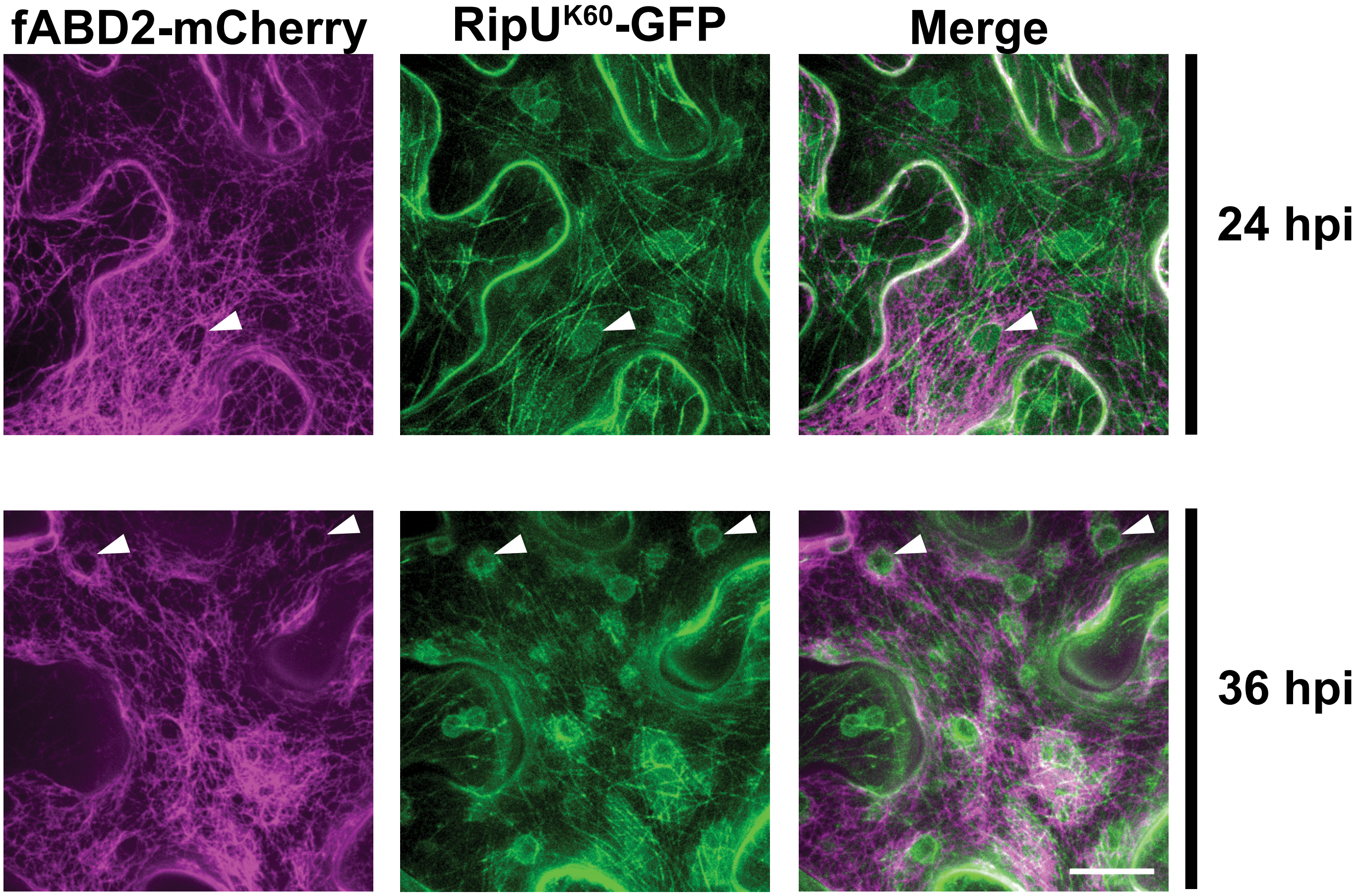

Supporting Fig 3. RipUK60-GFP and fABD2-mCherry surround intracellular components. Spinning disk confocal images at 24 (top) and 36 (bottom) hpi. The actin marker and RipU appear to surround an unidentified intracellular component. Arrows point to compartments.
