# Supplementary material for: A Ralstonia solanacearum type III effector alters the actin and microtubule cytoskeleton to promote bacterial virulence in plants
Source: PLoS Pathog. 2024 Dec 26;20(12):e1012814. doi: 10.1371/journal.ppat.1012814 (PMC11723619; doi:10.1371/journal.ppat.1012814)
Supplement: S4 Fig — (PDF) [file ppat.1012814.s004.pdf]

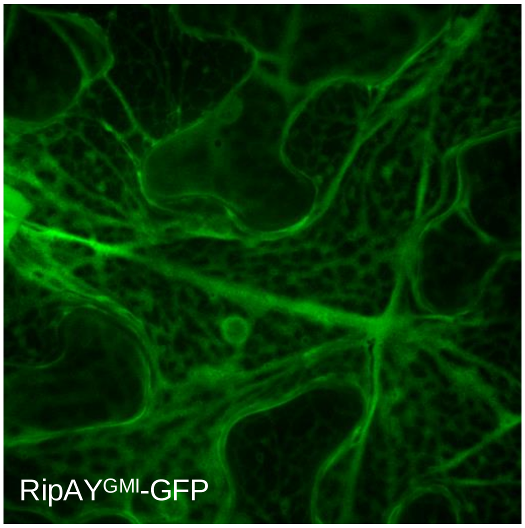

Supporting Fig 4. Spinning disk confocal maximum projection of RipAY^{GMI}-GFP 48 hpi after transient expression in *N. benthamiana* leaves.
