# Supplementary material for: A Ralstonia solanacearum type III effector alters the actin and microtubule cytoskeleton to promote bacterial virulence in plants
Source: PLoS Pathog. 2024 Dec 26;20(12):e1012814. doi: 10.1371/journal.ppat.1012814 (PMC11723619; doi:10.1371/journal.ppat.1012814)
Supplement: S5 Fig — Spinning disk confocal images of RipAYGMI:GFP and RipUK60:GFP co-infiltrated with either (A) actin reporter fABD2-mCherry or (B) microtubule reporter mTUB5-mCherry. Images were captured 48 hpi after agroinfiltration. Five to fifteen cells were measured at each infiltration site and the values were averaged as one biological sample (n). Three biological samples were quantified in each of two independent experiments. Each independent experiment is depicted as a different shape within each treatment. (C) Pearson’s colocalization analysis of RipU, RipAY and RipBD with fABD2. RipBD images are not shown but were performed as part of the same experiment. (D) Pearson’s colocalization analysis of RipU, RipAY and RipBD with mCherry. RipBD images are not shown but were performed as part of the same experiment. Letters indicate significance with a Tukey’s test after one-way ANOVA. (PDF) [file ppat.1012814.s005.pdf]

Supporting Figure 5

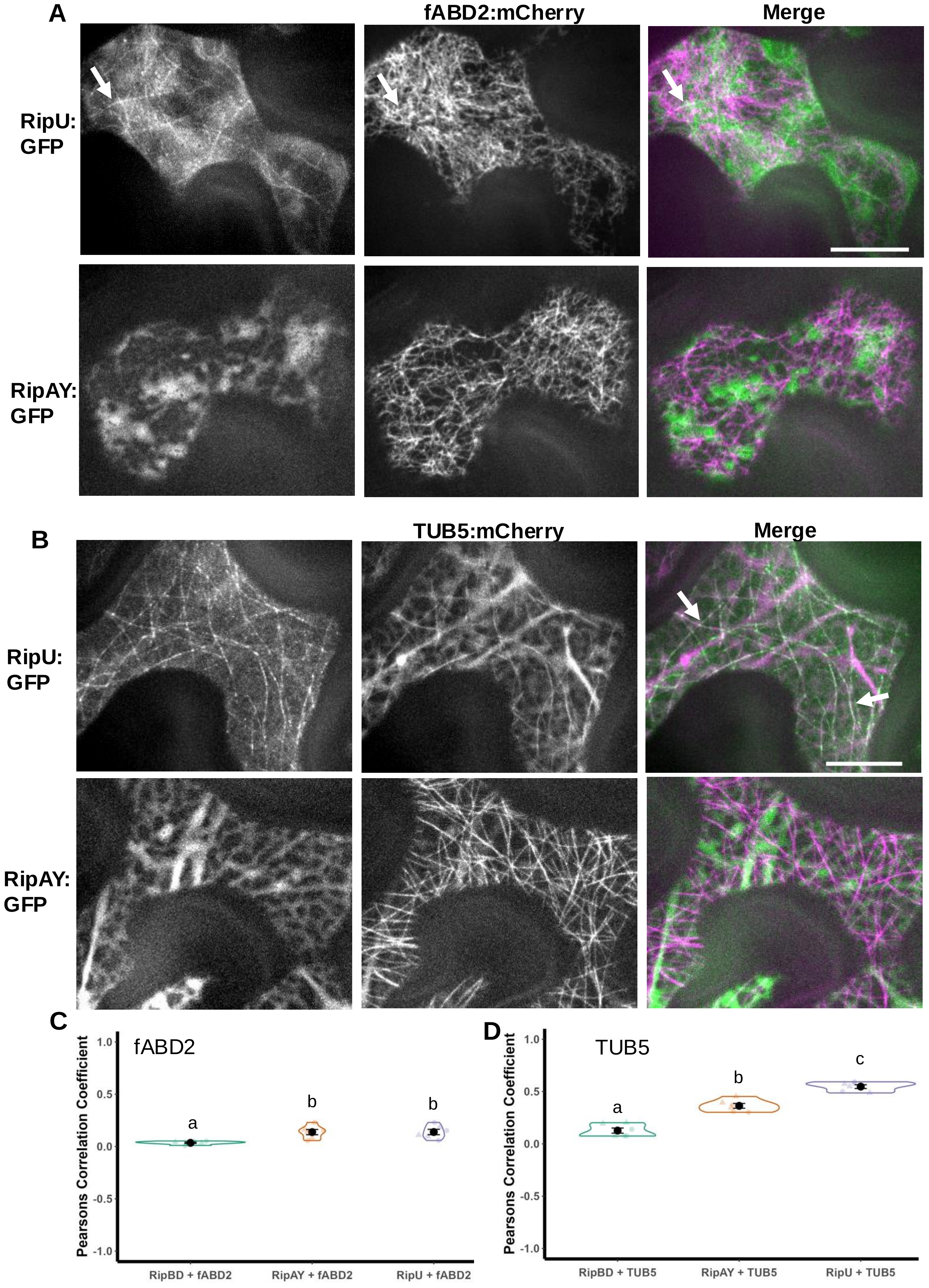

Supporting Figure 5 legend

**Supporting Fig 5. Co-localization analysis of RipU and the cytoplasmic and nuclear localized effector RipAY with the cytoskeleton 48 hpi in transient expression assays in *N. benthamiana* leaves.** Spinning disk confocal images of RipAY$^{GMI}$:GFP and RipU$^{K60}$:GFP co-infiltrated with either (A) actin reporter fABD2-mCherry or (B) microtubule reporter mTUB5-mCherry. Images were captured 48 hpi after agroinfiltration. Five to fifteen cells were measured at each infiltration site and the values were averaged as one biological sample (n). Three biological samples were quantified in each of two independent experiments. Each independent experiment is depicted as a different shape within each treatment. (C) Pearson's colocalization analysis of RipU, RipAY and RipBD with fABD2. RipBD images are not shown but were performed as part of the same experiment. (D) Pearson's colocalization analysis of RipU, RipAY and RipBD with mCherry. RipBD images are not shown but were performed as part of the same experiment. Letters indicate significance with a Tukey's test after one-way ANOVA.
