# Supplementary material for: A Ralstonia solanacearum type III effector alters the actin and microtubule cytoskeleton to promote bacterial virulence in plants
Source: PLoS Pathog. 2024 Dec 26;20(12):e1012814. doi: 10.1371/journal.ppat.1012814 (PMC11723619; doi:10.1371/journal.ppat.1012814)
Supplement: S6 Fig — (PDF) [file ppat.1012814.s006.pdf]

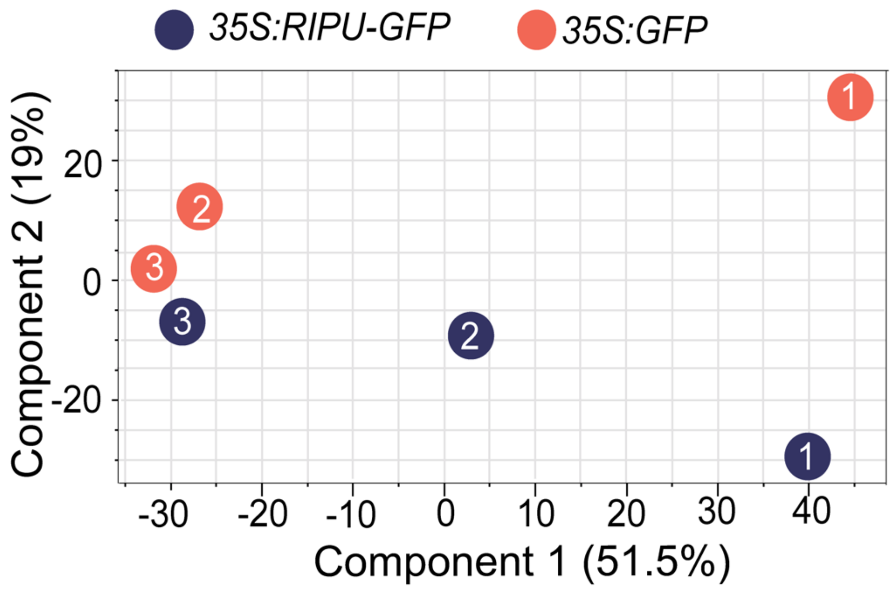

Supporting Fig 6: Principal component analysis (PCA) between the three Co-IP independent experiments between 35S:GFP and 35S:RipU:GFP.
