# Supplementary material for: A Ralstonia solanacearum type III effector alters the actin and microtubule cytoskeleton to promote bacterial virulence in plants
Source: PLoS Pathog. 2024 Dec 26;20(12):e1012814. doi: 10.1371/journal.ppat.1012814 (PMC11723619; doi:10.1371/journal.ppat.1012814)
Supplement: S7 Fig — (A) GO biological process categories related to the cytoskeleton that are enriched among downregulated genes in susceptible tomatoes in Meline et al. 2023 (53). (B) Genes in the categories in (A) and their closest Arabidopsis homolog identified from Phytozome 13. (PDF) [file ppat.1012814.s007.pdf]

A

Pathway	Enrichment FDR	No. Genes in Dataset	No. Genes in Pathway
CYTOSKELETON ORGANIZATION	0.00180401	11	159
ACTIN FILAMENT ORGANIZATION	0.007836419	6	56
ACTIN FILAMENT-BASED PROCESS	0.009984507	6	61
ACTIN CYTOSKELETON ORGANIZATION	0.009984507	6	61
ACTIN FILAMENT NETWORK FORMATION	0.027960286	2	4

B

Sol_ID	Protein	Arabidopsis Homolog
SOLYC01G111560	RAC-LIKE GTP-BINDING PROTEIN ARAC3	AT4G35020 (RAC3)
SOLYC02G068720	MEMBRANE-ASSOCIATED PROTEIN HEM	AT2G35110 (HEM1)
SOLYC09G010440	ACTIN-DEPOLYMERIZING FACTOR 1-RELATED	AT3G46010 (ADF1)
SOLYC10G078340	STOMATAL CLOSURE-RELATED ACTIN-BINDING PROTEIN 3	AT3G56480 (SCAB3)
SOLYC09G056170	FIMBRIN/PLASTIN	AT5G48460 (AtFIM2)
SOLYC06G083070	FIMBRIN-2-LIKE	AT5G48460 (AtFIM2)
SOLYC03G114760	PROTEIN MICROTUBULE BINDING PROTEIN 2C	AT5G08120 (MPB2C)
SOLYC04G081490	TUBULIN BETA-1 CHAIN-RELATED	AT1G75780 (TUB1)
SOLYC08G066050	AURORA KINASE	AT2G25880 (AUR2)
SOLYC03G116370	MICROTUBULE-ASSOCIATED PROTEIN RP/EB FAMILY MEMBER 1B	AT5G62500 (EB1)
SOLYC02G070440	KATANIN P60 ATPASE-CONTAINING SUBUNIT A-LIKE 2	AT2G34560 (AtCCP1)

Supporting Fig 7: Cytoskeleton-related Gene Ontology (GO) categories are enriched among down-regulated genes in a meta-analysis of *Ralstonia*-infected tomatoes. (A) GO biological process categories related to the cytoskeleton that are enriched among downregulated genes in susceptible tomatoes in Meline et al. 2023 (53). (B) Genes in the categories in (A) and their closest Arabidopsis homolog identified from Phytozome 13.
