# Supplementary material for: A Ralstonia solanacearum type III effector alters the actin and microtubule cytoskeleton to promote bacterial virulence in plants
Source: PLoS Pathog. 2024 Dec 26;20(12):e1012814. doi: 10.1371/journal.ppat.1012814 (PMC11723619; doi:10.1371/journal.ppat.1012814)
Supplement: S8 Fig — (A) Actin density (percent occupancy). (B) Microtubule number. Five to fifteen cells were measured at each infiltration site and the values were averaged as one biological sample (n). Three biological samples were quantified in each of two independent experiments. Each independent experiment is depicted as a different shape within each treatment. Letters indicate significance with a Tukey’s test after ANOVA. (PDF) [file ppat.1012814.s008.pdf]

Supporting Figure 8

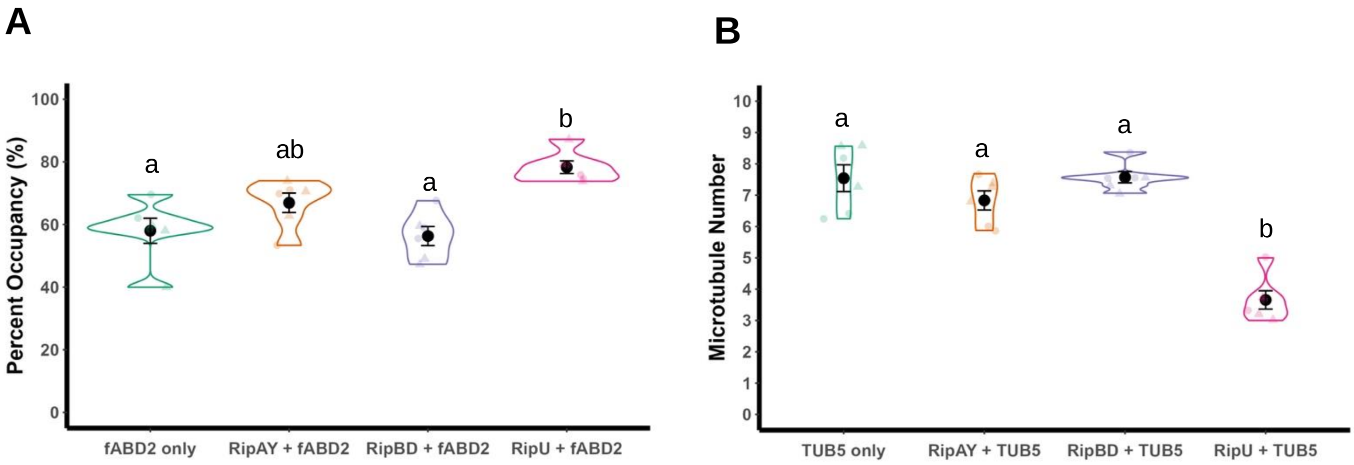

Supporting Fig 8 : Actin and microtubule organization 48 hpi after transient expression of RipAY, RipBD, RipU and fABD2 in *N. benthamiana* epidermal leaf cells. (A) Actin density (percent occupancy). (B) Microtubule number. For both (A) and (B) five to fifteen cells were measured at each infiltration site and the values were averaged as one biological sample (n). Three biological samples were quantified in each of two independent experiments. Each independent experiment is depicted as a different shape within each treatment. Letters indicate significance with a Tukey’s test after ANOVA.
